# Efficacy and safety of Wuhu oral liquid in treating acute soft tissue injuries: a multicenter, randomized, double-blind, double-dummy, parallel-controlled trial

**DOI:** 10.3389/fphar.2024.1335182

**Published:** 2024-02-23

**Authors:** Wen-Hao Zhu, Yi Shen, Yu Xiao, Qi Shi, Zhao-Xiang Fan, Yan-Qi Feng, Hong-Bo Wan, Bo Qu, Jun Zhao, Wei-Qiang Zhang, Guo-Hui Xu, Xue-Qun Wu, De-Zhi Tang

**Affiliations:** ^1^ Longhua Hospital, Shanghai University of Traditional Chinese Medicine, Shanghai, China; ^2^ Institute of Spine, Shanghai University of Traditional Chinese Medicine, Shanghai, China; ^3^ Shanghai University of Traditional Chinese Medicine, Shanghai, China; ^4^ Key Laboratory of Theory and Therapy of Muscles and Bones, Ministry of Education, Shanghai, China; ^5^ The First Affiliated Hospital of Chengdu Medical College, Chengdu, China; ^6^ Xi’an Hospital of Traditional Chinese Medicine, Xi’an, China; ^7^ Huadong Hospital Affiliated to Fudan University, Shanghai, China

**Keywords:** Chinese medicine, Wuhu oral liquid, acute soft tissue injury, multicenter, double-blind, parallel-controlled

## Abstract

**Background:** Wuhu Oral Liquid (WHOL) is a modified preparation derived from the famous Wuhu Powder, which has a long history of use in treating traumatic injuries. This preparation has anti-inflammatory and analgesic properties and accelerates recovery following acute soft tissue injuries.

**Aims:** To evaluate the efficacy and safety of WHOL in treating acute soft tissue injury associated with qi stagnation and blood stasis syndrome and to provide a basis for applying for the protection of varieties of Chinese medicine for WHOL.

**Methods:** This study was a randomized, controlled, double-blind, multicenter clinical trial in which Fufang Shang Tong Capsule (FFSTC) was selected as the control drug. A total of 480 subjects with acute soft tissue injury associated with qi stagnation and blood stasis syndrome were randomly divided into a test and control group in a 3:1 ratio. The duration of drug treatment was 10 days. The primary outcome was Visual Analogue Scale (VAS) score for pain (including pain at rest and pain on activity). Secondary outcomes included the disappearance time of the pain at rest and on activity; the curative effect of TCM syndrome and improvement in the individual symptoms of TCM (swelling, ecchymosis, and dysfunction); and changes in C-reactive protein (CRP) and interleukin-6 (IL-6) levels. Safety was assessed using vital signs, laboratory examinations, electrocardiograms, and physical examinations.

**Results:** Patient compliance was satisfactory in both groups (all between 80% and 120%). After 4 days of treatment, the WHOL group was superior to the FFSTC group in decreasing the VAS scores for pain at rest (−1.88 ± 1.13 vs*.* −1.60 ± 0.93, *p* < 0.05) and on activity (−2.16 ± 1.18 vs*.* −1.80 ± 1.07, *p* < 0.05). After 7 days of treatment, the WHOL group was superior to the FFSTC group in decreasing the VAS scores for pain on activity (−3.87 ± 1.60 vs*.* −3.35 ± 1.30, *p* < 0.01) and improving swelling (cure rate: 60.4% vs*.* 46.2%, *p* < 0.05; obvious effective rate: 60.7% vs*.* 47.0%, *p* < 0.05). After 10 days of treatment, the WHOL group was superior to the FFSTC group in decreasing the levels of CRP (−0.13 ± 2.85 vs*.* 0.25 ± 2.09, *p* < 0.05) and improving the TCM syndrome (cure rate: 44.1% vs*.* 30.8%, *p* < 0.05) and swelling (cure rate: 75.6% vs*.* 67.5%, *p* < 0.01; obvious effective rate: 75.6% vs*.* 68.4%, *p* < 0.05; effective rate: 77.0% vs*.* 71.8%, *p* < 0.05). The disappearance time of pain at rest was 8 days in both groups and 9 days on activity in both groups. In addition, there was no statistical difference between the incidence of adverse events (4.5% vs*.* 2.6%, *p* > 0.05) and adverse reactions (0.3% vs*.* 0%, *p* > 0.05) between the WHOL group and the FFSTC group. No serious adverse events occurred in either group, and no subjects were withdrawn because of adverse events.

**Conclusion:** WHOL relieves the symptoms caused by acute soft tissue injury associated with qi stagnation and blood stasis syndrome more rapidly than FFSTC, and it is effective and safe in the treatment of acute soft tissue injury. Future studies still need a larger sample size to verify its efficacy and safety.

**Clinical Trial Registration:** https:// www.chictr.org.cn/showproj.html?proj=149531, Identifier ChiCTR2200056411.

## 1 Introduction

Acute soft tissue injuries refer to a range of trauma syndromes caused by direct or indirect damage to soft tissues or skeletal muscles. These include acute injury to tissues such as muscles, ligaments, fascia, tendons, synovium, fat, and joint capsules, as well as peripheral nerves and blood vessels (Sloan, 2008). Acute soft tissue injuries represent a large proportion of cases seen in emergency medicine departments, constituting approximately 5%–10% of emergency department attendances in the United Kingdom (Handoll et al., 2007; Jones et al., 2020). In addition, the costs associated with acute soft tissue injuries, which may seem “minor,” are substantial, and approach $2 billion annually in Australia and over $100 million in New Zealand (Jones et al., 2020).

Acute soft tissue injuries are characterized by aseptic inflammation. The common clinical symptoms and signs of acute soft tissue injuries include pain, swelling, stasis, and dysfunction of the affected tissue (Jiang et al., 2020). The P.R.I.C.E principle or the P.O.L.I.C.E principle is commonly applied in modern medicine to treat acute soft tissue injuries. However, these principles cannot be used as standards for treatment because they do not apply to all types of acute soft tissue injuries (Bleakley, 2013). In addition, non-steroidal anti-inflammatory drugs (NSAIDs) are usually recommended for the pharmacological treatment of acute tissue injuries (Sloan, 2008; Jones et al., 2020). However, the use of NSAIDs is associated with several adverse reactions, among which gastrointestinal reactions are the most common. Other NSAID-associated adverse reactions include acute renal failure, cardiovascular adverse reactions, hemorrhagia, and bronchospasm (Jones et al., 2020). Therefore, the use of NSAIDs is restricted in patients with preexisting gastrointestinal disease, cardiovascular disease, and renal insufficiency, as well as the elderly (Wang, 2021). Traditional Chinese medicine (TCM) has shown great value in treating acute soft tissue injuries due to its efficacy, simplicity of operation, and few side effects (Chen et al., 2011; Wang et al., 2016; Huang et al., 2020).

TCM classifies acute soft tissue injury as “tendon injury.” The underlying pathogenesis involves qi stagnation and blood stasis. TCM theory suggests that the normal flow of blood in the vessels is propelled by Qi. When the movement of Qi is obstructed (Qi stagnation), blood flow loses its momentum and stagnates, forming blood stasis. Meanwhile, blood stasis can hinder the circulation of Qi and exacerbate Qi stagnation. Qi stagnation and blood stasis are mutually causal and generally coexist. The Qi stagnation and blood stasis syndrome is the pathological state of Qi stagnation and blood stasis coexisting (Huang et al., 2020). Following trauma, vessels in the injured area are damaged, obstructing the circulation of Qi and blood, resulting in stagnation of blood stasis in the vessels and poor circulation of Qi, leading to Qi stagnation and blood stasis syndrome.

WHOL is a liquid product prepared using the Wuhu Powder formula outlined in the Chinese Pharmacopoeia. The original prescription of Wuhu Powder dates back to the Qing Dynasty and has been used to treat traumatic injuries for more than 130 years. It has been recorded in all editions of the Chinese Pharmacopoeia. An experimental study demonstrated the remarkable analgesic and anti-inflammatory effects of the ethanolic extract of Wuhu Powder 30 years ago (Wang et al., 1990). There have also been reports of favorable outcomes in patients with acute soft tissue injuries who received treatment with Wuhu Powder (Lv and Rong, 2005). Notably, the use of Wuhu Powder in its powdered form has several drawbacks. These drawbacks include inconvenience in administration, poor bioavailability, and challenges in quality control (Deng et al., 2000). Jiangsu Jiuxu Pharmaceutical Group Co., Ltd. addressed the limitations of Wuhu Powder by developing a new dosage form (WHOL). A study (Deng et al., 2000) demonstrated that WHOL has superior and rapid analgesic and anti-inflammatory effects compared to Wuhu Powder.

This study aimed to evaluate the efficacy and adverse effects of WHOL in treating acute soft tissue injury with Qi stagnation and blood stasis using FFSTC as control. FFSTC has also been clinically proven effective in the treatment of acute soft tissue injury (Zheng et al., 2012; Du, 2016; Ge and Fan, 2017; Chang et al., 2021). Furthermore, the study provides a basis for applying for the protection of WHOL as an innovative TCM.

## 2 Materials and methods

### 2.1 Investigational medications

As a “Type A extract” (Heinrich et al., 2022), WHOL is composed of five botanical drugs. Through modern pharmaceutical technology, these five botanical drugs were mixed in different proportions, extracted, concentrated, and filtered to obtain WHOL. The quality control analysis of WHOL used high-performance liquid chromatography (HPLC) (see Supplementary Material for details). The proportion of botanical drugs contained in each standard dose (10 mL) of WHOL is as follows: *Angelica sinensis* (Oliv.) Diels [Apiaceae; Angelica sinensis radix]: 1.28 g; *Carthamus tinctorius* L. [Asteraceae; Carthami flos]: 1.28 g; *Saposhnikovia divaricata* (Turcz. ex Ledeb.) Schischk. [Apiaceae; Saposhnikoviae radix]: 1.28 g; *Arisaema erubescens* (Wall.) Schott [Araceae; Arisaematis rhizoma]: 1.28 g; and *Angelica dahurica* (Hoffm.) Benth. & Hook.f. ex Franch. & Sav. [Apiaceae; Angelicae dahuricae radix]:0.88 g. WHOL has been approved by the National Medical Products Administration (NMPA) (approval number: Z20184009). The oral liquid was manufactured by Jiangsu Jiuxu Pharmaceutical Group Co., Ltd. (Batch number: 20210701/20210601).

FFSTC, the control drug, is composed of eight botanical drugs, including *Rheum palmatum* L. [Polygonaceae; Rhei radix et rhizoma], *Bupleurum chinense* DC. [Apiaceae; Bupleuri radix], *Angelica sinensis* (Oliv.) Diels [Apiaceae; Angelica sinensis radix], *Prunus persica* (L.) Batsch [Rosaceae; Persicae semen], *Carthamus tinctorius* L. [Asteraceae; Carthami flos], *Corydalis yanhusuo* (Y.H.Chou & Chun C.Hsu) W.T.Wang ex Z.Y.Su & C.Y.Wu [Papaveraceae; Corydalis rhizoma], *Trichosanthes kirilowii* Maxim.[Cucurbitaceae; Trichosanthis radix], *Glycyrrhiza uralensis* Fisch. ex DC. [Fabaceae; Glycyrrhizae radix et rhizoma]. FFSTC has been approved by NMPA (approval number: Z20073054). The capsule was manufactured by Gansu Province Xifeng Pharmaceutical Co. Ltd. (Batch number: 105009/13210902).

The placebo in both test and control groups was composed of maltodextrin. Jiangsu Jiuxu Pharmaceutical Group Co., Ltd. provided all of the investigational medications.

### 2.2 Study design

This study was a multicenter, randomized, double-blind, parallel-controlled clinical trial. A total of 480 patients with acute soft tissue injury syndrome associated with qi stagnation and blood stasis were enrolled across 14 clinical trial centers from November 2021 to August 2022. This study was approved by the Clinical Research Ethics Committee of Longhua Hospital, Shanghai University of TCM (approval number: 2021LCSY114) and the 13 other sites. The protocol was also registered in the Chinese Clinical Trial Registry (registration number ChiCTR2200056411). All participants signed an informed consent form. Plant use in this study complied with the relevant laws and regulations of the national and local governments to protect biodiversity. The study was conducted per the requirements of the CONSORT Extension for Chinese Herbal Medicine Formulas (Cheng et al., 2017), the Code for Quality Management of Drug Clinical Trials (National Medical Products Administration, 2020), the Guidelines for the Protection of Varieties of Chinese Medicine (National Medical Products Administration, 2009), the Guiding Principles for Clinical Research of New Chinese Medicines (Zheng, 2002), and the Declaration of Helsinki. Each hospital had a designated investigator responsible for the quality of the clinical trial, and standardized training was provided to the designated investigator and participating investigators at each hospital before the start of the trial.

### 2.3 Diagnostic, inclusion and exclusion criteria

Diagnostic criteria of Western medicine: 1) Obvious history of trauma; 2) Obvious pressure and pain at the injured site, skin bruising, petechiae, localized swelling, and, in severe cases, subcutaneous hematoma and limb dysfunction; 3) X-ray examination: mainly used for checking whether there are fracture, dislocation, and osteopathy at the injured site, which has certain reference value for assessing tendon, ligament rupture, and cartilage injuries. Formulated with reference to the Guiding Principles for Clinical Research of New Chinese Medicines (Zheng, 2002) and the Expert Consensus on Diagnosis, Treatment, and Pain Management of Acute Closed Soft Tissue Injuries (Wang, 2021).

Diagnostic criteria of TCM (Li et al., 2022; Zhu et al., 2022; Li et al., 2023): Qi stagnation and blood stasis syndrome: 1) Primary symptoms: localized, stabbing pain in a definite place or with impaired mobility; 2) Secondary symptoms: localized swelling, bruises, and petechiae or hematoma; 3) Tongue: purplish and dark tongue or petechiae; 4) Pulse: stringy and astringent pulse. These primary symptoms are essential, with or without other symptoms. Formulated with reference to the Guiding Principles for Clinical Research of New Chinese Medicines (Zheng, 2002) and the Chinese Medicine Industry Standard of the People’s Republic of China-Diagnostic Efficacy Criteria for TCM Evidence (ZY/T001.9).

Inclusion criteria: 1) Met the diagnostic criteria for acute closed soft tissue injuries in “*The Expert Consensus on Diagnosis, Treatment, and Pain Management of Acute Closed Soft Tissue Injuries*” (Wang, 2021), including injuries to subcutaneous tissues or muscles, tendons, fascia, ligaments, and joint capsules attached to the skeletal structure. The skin and mucous membranes at the injury site should remain intact without an open wound; 2) Met the diagnostic criteria of acute soft tissue injury in Western medicine; 3) Met the diagnostic criteria of qi stagnation and blood stasis syndrome in TCM; 4) Duration of soft tissue injury ≤48 h; 5) Visual analogue scale (VAS) score at rest >3 points, VAS score on activity <9 points; 6) Aged 18–65, regardless of gender; 7) Participants who gave written informed consent.

Exclusion criteria: 1) Soft tissue injury with fracture, bone fissure, open wound, or complete rupture of muscle, tendon, and ligament; 2) Soft tissue injury sites ≥2; 3) Presence of comorbid inflammatory pain diseases such as rheumatoid arthritis, psoriatic arthritis, and gout; 4) Participants who had taken NSAIDs, similar medicines, corticosteroids, or antibiotics within a week, or had received alternative therapies such as acupuncture, physiotherapy, or manipulation before the first dose; 5) Severe heart disease, renal failure, hematological disease, or abnormal liver and kidney function; 6) Pregnant, suspected pregnant, or lactating women; 7) Participants with physical or mental diseases that affect cooperation, or serious diseases such as tumors that affect survival; 8) Suspected or proven history of alcohol or drug abuse; 9) Known allergy to trial drug, alcohol, or protocol-prescribed emergency medication; 10) Participants who engaged in high-altitude, high-risk work, or professional driver.

### 2.4 Randomization and control

According to the requirements of the Guiding Principles for the Protection of Varieties of TCM issued by the National Medical Products Administration (NMPA), which states that the selection of parallel control drugs should follow the principles of “widely recognized, of the same category, and based on excellence.” FFSTC is recommended by the expert consensus of the Chinese Academy of TCM for the treatment of stasis syndrome in acute chest contusion injuries or blood stasis and Qi stagnation syndrome in acute soft tissue injuries. Therefore, the use of FFSTC in the treatment of Qi stagnation and blood stasis syndrome in acute soft tissue injuries is in line with the principle of “widely recognized.” Both FFSTC and WHOL are purely Chinese medicine preparations that are used to promote blood circulation and remove blood stasis, and have similar therapeutic effects, thus meeting the principle of “of the same category.” FFSTC has been on the market for more than 10 years and has been widely used in departments such as orthopedics, thoracic surgery, emergency medicine, and pain management. FFSTC’s clinical efficacy in treating soft tissue injuries has been confirmed, and it is considered a high-quality product among similar drugs, conforming to the principle of “based on excellence.” Therefore, FFSTC was selected as the parallel control drug.

Block randomization was applied. Random allocation codes (code range 001–480) were generated using the SAS software. The recruitment capacity for each trial center was determined before the start of the trial. Furthermore, a statistician evaluated and designed the appropriate number of blocks and block lengths based on the number of subjects expected to be recruited at each trial center (finalized number of blocks = 120 and block length = 4). Code segments were assigned to each center in multiples of 4 based on the number of subjects expected to be recruited at each trial center. The different code segments were sent to each trial center after the allocation, with corresponding treatment kits. During the trial, cases were deployed based on the progress of enrollment in each center to ensure that all centers completed the trial at the same time. The randomization sequence was designed by a statistician who did not participate in the trial. After obtaining the consent of the subjects, the research physicians overseeing recruitment at each site contacted the appointed drug administrator at the respective site. This drug administrator, kept independent of the recruitment process, was contacted by phone.

### 2.5 Blinding

The trial was a double-blind study and ensured that neither the subjects, investigators, supervisors, nor data analysts were aware of the distribution of the treatment drugs. The sponsor provided the test drug, with uniform internal and external packaging. Because the dosage forms of both test and control drugs were different, the oral liquid placebo and capsule placebo were made separately to ensure the reliability of the blinding method. Patients in the test group received WHOL and FFSTC placebo, while those in the control group received FFSTC and WHOL placebo. The appearance, daily frequency of administration, and dose of the two groups of drugs were consistent. Furthermore, the drugs were packaged in sealed, opaque boxes with the same label. The label outside each box indicated the clinical study drug (WHOL), the same usage and dosage, the storage conditions, and the expiry date. Appointed clinical trial drug administrators at each site were responsible for receiving, storing, distributing, and retrieving surplus drugs and used drug cartridges.

The blinding envelope contained the specific treatment group (test group, control group) corresponding to each subject. The blinding envelopes were sealed separately, in duplicate, and one copy was kept at the team leader’s office and the other in the applicant’s office. Each test drug was assigned a unique code, and corresponding emergency letters were provided for each group to break the blind in the event of an emergency. The emergency letters were sealed and sent, along with the corresponding coded test drugs, to each clinical trial center. The centers were responsible for the preservation of the letters and did not open the letters unless it was necessary. In the event of an emergency, such as a serious adverse event or when a subject needed to be rescued, it was necessary to know what treatment the subject was receiving. Once the blind was broken for any particular subject, that subject was withdrawn from the trial and treated as a dropped case, and the investigator recorded the reason for withdrawal in a case report form. After all the case report forms were entered into a database and challenged, verified, and blinded for review, the data were locked. The unblinding was carried out by the staff member who kept the blinded envelopes.

### 2.6 Intervention and efficacy evaluation

The study period consisted of visits V1 to V5. V1 was at day 0 for screening and baseline laboratory examinations, V2 was at 0.5 h of drug administration, V3 was at day 4, V4 was at day 7, and V5 was at day 10. Eligible subjects were assigned to test or control groups. The test and control drugs were administered orally, and the doses administered were: Oral Liquid, 10 mL twice daily; Capsule, 3 capsules thrice daily. The treatment duration was 10 days. NSAIDs were administered when a subject continued to experience pain that they could not tolerate after 24 h of receiving the assigned treatment. Subjects were immediately withdrawn in instances where the pain persisted after at least two doses of NSAIDs were administered, and medical attention was promptly sought. All relevant drug information, including drug name, dosage, indication, frequency, and duration of use, were documented on the case report form to allow for comprehensive analysis and reporting. Compliance was calculated as the actual dosage/prescribed dosage × 100%, and compliance between 80% and 120% was considered satisfactory; compliance <80% or >120% was considered poor.

The primary efficacy indicator was VAS scores, including reductions in VAS scores for pain at rest and pain on activity at 0.5 h, 4 days, 7 days, and 10 days after treatment. Evaluation method: Change values = post-treatment (rest/activity) pain VAS score—pre-treatment (rest/activity) pain VAS score. The secondary efficacy indicators were: 1) Disappearance time of the pain at rest and on activity, Evaluation method: time (days) when (rest/activity) pain VAS score = 0; 2) Curative effect of TCM syndrome at 4, 7, and 10 days after treatment, Evaluation method: Curative effect of TCM syndrome = (pre-treatment TCM syndrome score - post-treatment TCM syndrome score)/pre-treatment TCM syndrome score × 100%. Results ≥95%, ≥70%, ≥30%, and <30% corresponded to cured, significantly effective, effective, and ineffective, respectively; 3) Individual symptoms of TCM syndrome, including improved swelling, ecchymosis, and dysfunction at 4, 7, and 10 days after treatment, Evaluation method: Disappearance of symptoms or signs = cured; Significant improvement in symptoms or signs (from severe to mild) = obvious effective; Improvement in symptoms or signs (from severe to moderate, or moderate to mild) = effective; No change or insignificant reduction in symptoms or signs, or aggravation = ineffective; 4) Levels of C-reactive protein (CRP) and interleukin (IL)-6 before and 10 days after treatment, Evaluation method: Change values = post-treatment (CRP/IL-6) measured value—pre-treatment (CRP/IL-6) measured value. The details of the TCM syndrome score and the assessment of the severity of pain, swelling, and dysfunction are presented in Table 1.

**TABLE 1:** TCM syndrome Scoring Scale*.

Primary symptom	Normal (0 score)	Mild (2 scores)	Moderate (4 scores)	Severe (6 scores)
Pain at rest (VAS score)	0	≤3	3–7	≥7
Pain on activity (VAS score)	0	≤3	3–7	≥7
Dysfunction	None	Mildly restricted, able to perform normal activities	Significantly limited, but still able to care for themselves	Loss of activity function
**Secondary symptom**	**Normal (0 score)**	**Mild (1 score)**	**Moderate (2 scores)**	**Severe (3 scores)**
Swelling	None	Light swelling, no obvious indentation when pressing the swollen area with the finger	Swelling is obvious, but skin texture is still smooth, press the swollen part with a finger, and there can be obvious depression	Significant swelling, elongated skin texture, tense shiny skin, and even tense blisters
Ecchymosis	None	Ecchymosis pale purple, area <2 × 2 cm	Ecchymosis purple, area ≥2 × 2 cm but <4 × 4 cm	Ecchymosis dark purple, area ≥4 × 4 cm
**Tongue and Pulse**	**Normal (0 score)**	**Mild (1 score)**	**Other (no score)**	
Tongue color	Light red	Dark purple or ecchymosis	Other ( )	
Pulse condition	Normal pulse	Wiry and unsmooth pulse	Other ( )	

*The TCM, syndrome score is the sum of the 3 primary symptom scores, the 2 secondary symptom scores, and the tongue and pulse scores. The score for the primary symptom is twice as high as the score for the secondary symptom and the tongue and pulse.

### 2.7 Safety assessment

Safety was assessed using vital signs, laboratory examinations, electrocardiograms, and physical examinations. The vital signs included body temperature, respiratory rate, pulse, and blood pressure. The laboratory examinations included complete blood count (white blood cells, red blood cells, hemoglobin, and platelets); urine tests (erythrocytes, leukocytes, glucose, and protein); liver function (alanine transaminase [ALT], aspartate transaminase [AST], alkaline phosphatase [ALP], total bilirubin, and gamma-glutamyl transpeptidase); and renal function (SCr and blood urea nitrogen). A 12-lead electrocardiogram was performed to assess cardiac activity and function. A thorough physical examination was conducted, including an assessment of the head, neck, chest, abdomen, and skin of the extremities for any signs of allergic reactions.

When an adverse event occurred, the investigators carefully observed and evaluated the association between the adverse event and the test drug, the severity of the adverse event, and the outcome of the adverse event. At the same time, necessary interventions such as dose adjustment and temporary dosing interruption were made, as well as a decision to withdraw the subject from the trial. The severity of adverse events was graded. Mild adverse events were tolerated by the subject, did not affect treatment, did not require special treatment, and had no effect on recovery; moderate adverse events were intolerable to the subject, required withdrawal or special treatment, and had a direct impact on recovery; and severe adverse events were life-threatening, fatal, or disabling to the subject, required immediate withdrawal or emergency treatment.

### 2.8 Sample size and statistical analysis

According to the Guiding Principles for the Protection of Varieties of TCM (National Medical Products Administration, 2009), the sample size needed to meet statistical requirements, with the test group typically having not less than 300 subjects. Under the premise of meeting the statistical requirements, the control group was generally designed to contain 1/3 of the number of subjects in the test group. Thus, a minimum of 400 subjects were needed. Given a 20% drop-off rate, a total of 480 subjects were included, of which 360 were in the test group and 120 were in the control group. In addition, the guideline requires that when drugs are used to treat multiple conditions, the number of cases for the primary condition in the test group must generally not be less than 60 cases. Therefore, acute soft tissue injury of the ankle joint was considered the primary condition in this trial, and the test group comprised not less than 60 cases.

According to the intention-to-treat (ITT) principle in the statistical principles for clinical trials (ICH E9) (JA, 1999), the full analysis set (FAS) (defined as subjects who received at least one dose of the therapeutic drug and had the corresponding efficacy evaluation) was used to evaluate the primary and secondary efficacy indicators in superiority trials. Adverse event assessment was based on the safety analysis set (SS) (defined as subjects who received at least one dose of the drug). Quantitative data are presented as mean ± standard deviation (SD) or median, and two-tailed Student’s t-test or Wilcoxon-Mann-Whitney test were used to test for differences between groups before and after treatment. For normally distributed data, the two-tailed Student’s t-test was used. Otherwise, the Wilcoxon-Mann-Whitney test was adopted. Qualitative data were described using frequency or composition ratio, and chi-square and Fisher’s exact tests were used to analyze the differences between the two groups of qualitative data results. Missing observations were imputed using the last-observation-carried-forward (LOCF) method, where the last value observed before dropout was used as the outcome for participants who withdrew from the trial prematurely. All tests were two-sided, and *p* < 0.05 was considered significant. All statistical analyses were performed using SAS 9.4 (SAS Institute, Cary, NC, United States).

## 3 Results

### 3.1 Baseline characteristics

**FIGURE 1:**
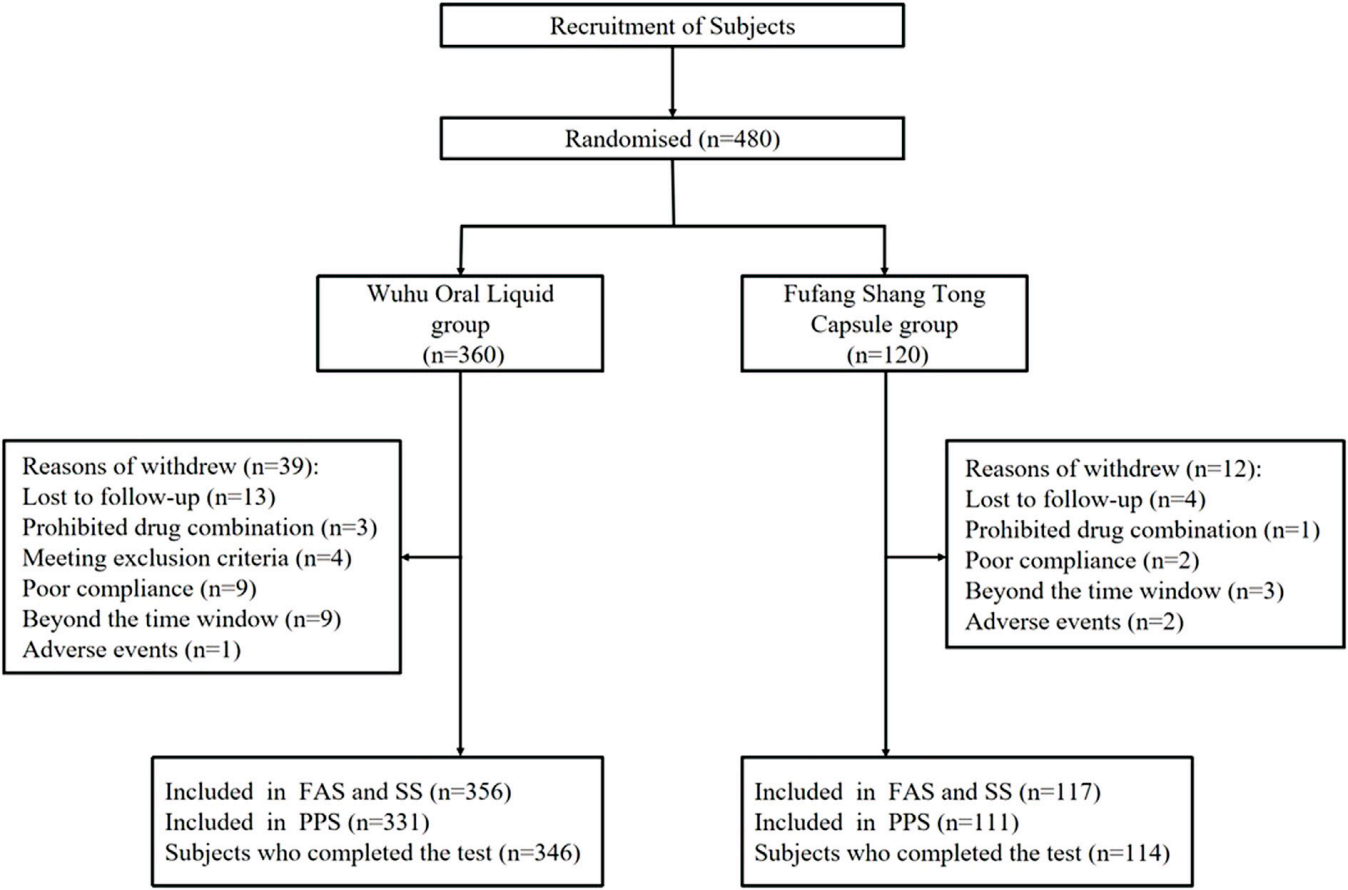
Study flowchart.

**FIGURE 2:**
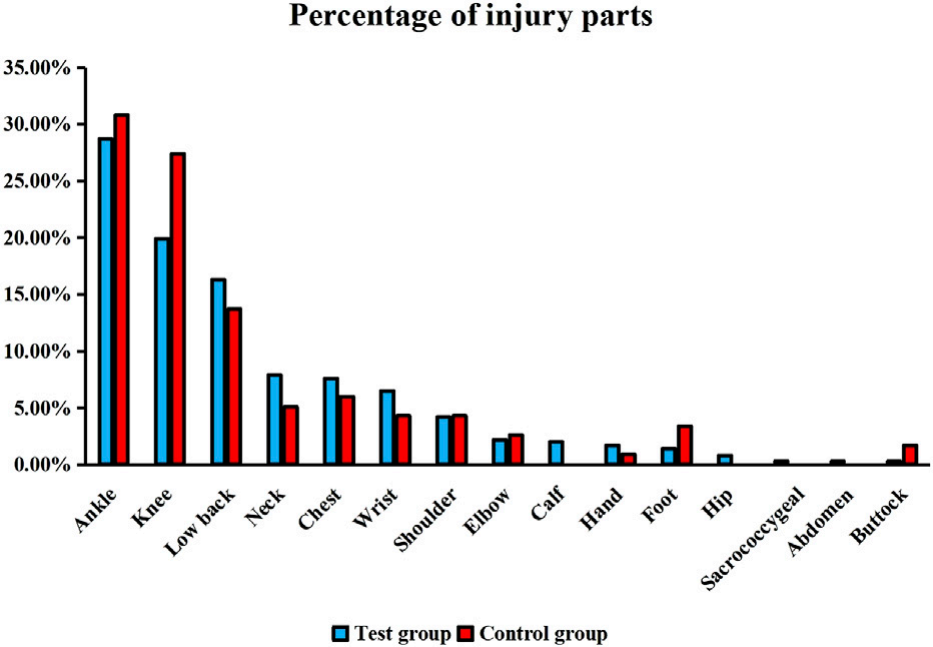
Percentage of injury sites.

This clinical trial was conducted at 14 sites, and a total of 480 subjects were enrolled: 360 in the test group and 120 in the control group. However, 460 subjects completed the trial: 114 (95.00%) in the control group and 346 (96.10%) in the test group (Figure 1). Subject characteristics are presented in Table 2. There were no statistical differences between the two groups in terms of demographic characteristics, past medical history, medication for other diseases, allergy history, and injured part (*p* > 0.05). The percentages of injury sites are presented in Figure 2. Notably, the medication adherence was 92.17% ± 6.97% in the test group and 92.73% ± 6.77% in the control group, and the difference in medication adherence between these two groups was not statistically significant. Meanwhile, the overall medication adherence in both groups was satisfactory and ranged from 80% to 120% (Table 3).

**TABLE 2:** Comparison of subject characteristics at baseline.

Characteristics	Test group (*n* = 356)	Control group (*n* = 117)	*p*-values
Age (years, $\bar{\chi }$ ± s)			
Mean (SD)	37.14 ± 13.44	37.20 ± 11.98	0.529
Min, Max	16.00–65.00	15.00–64.00	
Median (Q3-Q1)	34 (25.00–49.00)	34 (27.00–48.00)	
**Sex (%)**			
Men	169 (47.5)	52 (44.4)	0.569
Women	187 (52.5)	65 (55.6)	
**Nationality (%)**			
Han ethnic	339 (95.2)	109 (93.2)	0.387
Non-Han ethnic	17 (4.8)	8 (6.8)	
**Marital status (%)**			
Married	216 (60.7)	77 (65.8)	0.321
Others	140 (39.3)	40 (34.2)	
**Occupation (%)**			
Mental labor	246 (69.1)	79 (67.5)	0.643
Physical labor	92 (25.8)	34 (29.1)	
Others	18 (5.1)	4 (3.4)	
**Height (cm,** $\bar{\chi }$ **± s)**	166.60 ± 8.18	166.52 ± 8.19	0.934
**Weight (kg,** $\bar{\chi }$ **± s)**	63.72 ± 10.27	64.96 ± 11.52	0.595
**BMI (kg/m** ^**2**^ $\bar{\chi }$ **± s)**	22.87 ± 2.65	23.32 ± 2.98	0.143
**Past medical History (%)**	45 (12.6)	18 (15.4)	0.449
**Medication for other diseases (%)**	15 (4.2)	7 (6.0)	0.430
**Allergy history (%)**	4 (1.1)	3 (2.6)	0.372
**Injured part (%)**			0.532
Ankle	102 (28.7)	36 (30.8)	
Knee	71 (19.9)	32 (27.4)	
Low back	58 (16.3)	16 (13.7)	
Neck	28 (7.9)	6 (5.1)	
Chest	27 (7.6)	7 (6.0)	
Wrist	23 (6.5)	5 (4.3)	
Shoulder	15 (4.2)	5 (4.3)	
Elbow	8 (2.2)	3 (2.6)	
Calf	7 (2.0)	0 (0)	
Hand	6 (1.7)	1 (0.9)	
Foot	5 (1.4)	4 (3.4)	
Hip	3 (0.8)	0 (0)	
Sacrococcygeal	1 (0.3)	0 (0)	
Abdomen	1 (0.3)	0 (0)	
Buttock	1 (0.3)	2 (1.7)	

Statistics: *p*-values were calculated for continuous outcomes with the Wilcoxon-Mann-Whitney test; The chi-square test and Fisher’s exact test were performed for categorical outcomes; Injury parts were calculated using a Monte Carlo Estimate of the exact test.

**TABLE 3:** Medication adherence.

Category	Test group (%)	Control group (%)	P- values
Overall medication adherence			
Mean ± SD	92.17 ± 6.97	92.7 3 ± 6.77	0.703
Min—Max	45.00–120.00	70.89–120.00	
Q1–Q3	88.34–95.84	88.34–97.50	
Median	91.67	92.50	

Statistics: *p*-values were calculated with Wilcoxon–Mann–Whitney test.

### 3.2 Primary indicators

#### 3.2.1 Pain at rest

**FIGURE 3:**
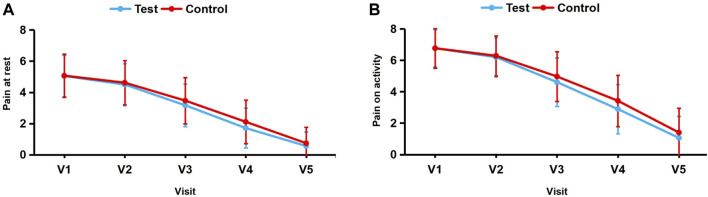
**(A, B)** Changes in mean VAS scores for pain at rest and on activity from baseline (visit 1) to visit 5.

The VAS scores for pain at rest at the injury site were 5.05 ± 1.33 in the test group and 5.07 ± 1.38 in the control group before treatment, with no statistically significant difference (*p* > 0.05). Figure 3A presents the VAS score changes for pain at rest in the test and control groups at each follow-up visit. Compared to baseline, the VAS scores for pain at rest at 0.5 h, 4 days, 7 days, and 10 days after treatment decreased by 0.55 ± 0.65, 1.88 ± 1.13, 3.34 ± 1.58, and 4.49 ± 1.54, respectively, in the test group and by 0.56 ± 0.65, 1.60 ± 0.93, 2.96 ± 1.29, and 4.33 ± 1.40, respectively, in the control group (Table 4).

**TABLE 4:** Measured values (MV) and change values (CV) relative to baseline in VAS scores for pain at rest and on activity at different follow-up times^a^.

Category	Test group (N = 356)		Control group (N = 117)		*p*-values
**Pain at rest**	MV	CV	MV	CV	
V1 (Baseline)	5.05 ± 1.33		5.07 ± 1.38		0.859
V2 (0.5 h)	4.50 ± 1.35	−0.55 ± 0.65	4.62 ± 1.41	−0.44 ± 0.58	0.380
V3 (Day 4)	3.17 ± 1.36	−1.88 ± 1.13	3.47 ± 1.49	−1.60 ± 0.93	0.031
V4 (Day 7)	1.71 ± 1.28	−3.34 ± 1.58	2.11 ± 1.39	−2.96 ± 1.29	0.082
V5 (Day 10)	0.56 ± 0.91	−4.49 ± 1.54	0.74 ± 1.03	−4.33 ± 1.40	0.365
**Pain on activity**	MV	CV	MV	CV	
V1 (Baseline)	6.77 ± 1.18		6.77 ± 1.26		0.929
V2 (0.5 h)	6.20 ± 1.25	−0.57 ± 0.69	6.29 ± 1.28	−0.47 ± 0.68	0.865
V3 (Day 4)	4.61 ± 1.54	−2.16 ± 1.18	4.97 ± 1.59	−1.80 ± 1.07	0.011
V4 (Day 7)	2.89 ± 1.57	−3.87 ± 1.60	3.42 ± 1.63	−3.35 ± 1.30	0.082
V5 (Day 10)	1.06 ± 1.37	−5.71 ± 1.63	1.40 ± 1.54	−5.37 ± 1.60	0.101

^a^ Data are presented as Mean ± SD, Change values (CV), Measured values (MV)—Baseline values at different follow-up times.

Statistics: *p*-values were calculated with Wilcoxon–Mann–Whitney test. Differences at baseline were compared based on measured values (MV) in both groups, while differences between both groups at different follow-up time points were compared based on change values (CV).

The test group exhibited significantly greater reductions in scores compared to the control group after 4 days of treatment (*p* < 0.05), indicating that WHOL was more effective in relieving pain at rest at the injury site than FFSTC. The difference between the two groups at 0.5 h, 7 days, and 10 days of treatment was not statistically significant (*p >* 0.05), suggesting that the efficacy of the two groups in relieving pain at rest at the injury site was not significantly different at 0.5 h, 7 days, and 10 days of treatment.

#### 3.2.2 Pain on activity

Before treatment the VAS scores for pain on activity at the injury site were 6.77 ± 1.18 in the test group and 6.77 ± 1.26 in the control group, with no statistically significant difference (*p* > 0.05). Figure 3B presents the VAS score changes for pain on activity in the test and control groups at each follow-up visit. Compared to baseline, the VAS scores for pain on activity at 0.5 h, 4 days, 7 days, and 10 days after treatment decreased by 0.57 ± 0.69, 2.16 ± 1.18, 3.87 ± 1.60, and 5.71 ± 1.63, respectively, in the test group and by 0.47 ± 0.68, 1.80 ± 1.07, 3.35 ± 1.30, and 5.37 ± 1.60, respectively, in the control group (Table 4).

The test group exhibited significantly greater reductions in VAS scores compared to the control group on days 4 and 7 (*p* > 0.05), suggesting that the effectiveness of WHOL in relieving the pain on activity at the injury site was superior to FFSTC at 4 and 7 days of treatment. However, there was no significant difference between the two groups in relieving the pain on activity at the injury site at 0.5 h and 10 days of treatment (*p* > 0.05).

### 3.3 Secondary indicators

#### 3.3.1 Disappearance time of the pain at rest and pain on activity

The median disappearance time of pain at rest was 8 days in both groups, and the median disappearance time of pain on activity was 9 days in both groups (*p* > 0.05) (Table 5).

**TABLE 5:** Disappearance time of the pain at rest and on activity.

	Test group (day)	Control group (day)	*p*-values
**Pain at rest**	8.00	8.00	0.179
**Pain on activity**	9.00	9.00	0.514

Statistics: The disappearance time of pain was skewness distribution and presented by a median. *p*-values were calculated with Wilcoxon–Mann–Whitney test.

#### 3.3.2 Curative effect of TCM syndrome

The TCM syndrome scores of the test group and control group were 14.26 ± 2.55 and 14.65 ± 2.68 (*p* > 0.05), respectively. The curative effect of TCM syndrome at days 4, 7, and 10 was analyzed, and the cure rate was 44.1% in the test group and 30.8% in the control group at day 10 (*p* < 0.05). Resultantly, it was suggested that the curative effect of WHOL in improving qi stagnation and blood stasis syndrome was superior to FFSTC after 10 days of treatment. However, the difference in total effective rate between the two groups was not statistically significant (*p* > 0.05) (Table 6).

**TABLE 6:** Curative effect of TCM syndrome.

Category		Test group	Control group	*p*-values
TCM syndrome	Efficacy			
N (Missing)		356 (0)	117 (0)	
V3 (Day 4)	Cured	5 (1.4%)	0 (0.0%)	0.339
	Obvious effective	7 (2.0%)	0 (0.0%)	0.201
	Effective	144 (40.4%)	49 (41.9%)	0.785
V4 (Day 7)	Cured	18 (5.1%)	1 (0.9%)	0.055
	Obvious effective	93 (26.1%)	20 (17.1%)	0.047
	Effective	317 (89.0%)	104 (88.9%)	0.964
V5 (Day 10)	Cured	157 (44.1%)	36 (30.8%)	0.010
	Obvious effective	249 (69.9%)	76 (65.0%)	0.301
	Effective	345 (96.9%)	112 (95.7%)	0.257

Statistics: Chi-square test or Fisher’s exact test was performed for curative effect analysis.

#### 3.3.3 Improvement in individual symptoms

There were no significant differences in the severity of dysfunction (2.79 ± 0.99 vs*.* 2.90 ± 1.03, *p* > 0.05), swelling (1.16 ± 0.38 vs*.* 1.25 ± 0.46, *p* > 0.05), and ecchymosis (1.28 ± 0.45 vs*.* 1.26 ± 0.48, *p* > 0.05) between the two groups before treatment. After 7 and 10 days of treatment, the test group exhibited a higher cure rate and obvious effective rate compared to the control group. The effective rate of the test group was also superior to that of the control group after 10 days of treatment. The differences were statistically significant (*p* < 0.05), suggesting that the effectiveness of WHOL in relieving swelling in the test group was superior to that of FFSTC after 7 and 10 days of treatment. In addition, there was no significant difference between the two groups in the improvement of dysfunction and ecchymosis (Table 7).

**TABLE 7:** Improvement in individual symptoms.

Category		Test group	Control group	*p*-values
Dysfunction	Efficacy			
N (Missing)		348 (8)	115 (2)	
V3 (Day 4)	Cured	49 (13.8%)	17 (14.5%)	0.852
	Obvious effective	49 (13.8%)	17 (14.5%)	0.852
	Effective	158 (44.4%)	57 (48.7%)	0.438
V4 (Day 7)	Cured	172 (48.3%)	53 (45.3%)	0.533
	Obvious effective	173 (48.6%)	54 (46.2%)	0.607
	Effective	263 (73.9%)	93 (79.5%)	0.235
V5 (Day 10)	Cured	306 (86.0%)	96 (82.1%)	0.243
	Obvious effective	306 (86.0%)	96 (82.1%)	0.243
	Effective	327 (91.9%)	104 (88.9%)	0.194
**Swelling**				
N (Missing)		292 (64)	96 (21)	
V3 (Day 4)	Cured	89 (25.0%)	21 (17.9%)	0.105
	Obvious effective	89 (25.0%)	21 (17.9%)	0.105
	Effective	120 (33.7%)	35 (29.9%)	0.421
V4 (Day 7)	Cured	215 (60.4%)	54 (46.2%)	0.001
	Obvious effective	216 (60.7%)	55 (47.0%)	0.002
	Effective	239 (67.1%)	71 (60.7%)	0.101
V5 (Day 10)	Cured	269 (75.6%)	79 (67.5%)	0.005
	Obvious effective	269 (75.6%)	80 (68.4%)	0.013
	Effective	274 (77.0%)	84 (71.8%)	0.045
**Ecchymosis**				
N (Missing)		163 (193)	57 (60)	
V3 (Day 4)	Cured	32 (9.0%)	11 (9.4%)	0.956
	Obvious effective	32 (9.0%)	11 (9.4%)	0.956
	Effective	55 (15.4%)	18 (15.4%)	0.765
V4 (Day 7)	Cured	105 (29.5%)	35 (29.9%)	0.684
	Obvious effective	105 (29.5%)	36 (30.8%)	0.865
	Effective	132 (37.1%)	45 (38.5%)	0.739
V5 (Day 10)	Cured	138 (38.8%)	47 (40.2%)	0.488
	Obvious effective	138 (38.8%)	47 (40.2%)	0.488
	Effective	152 (42.7%)	53 (45.3%)	0.519

Statistics: Chi-square test or Fisher’s exact test was performed for curative effect analysis.

#### 3.3.4 Levels of CRP and IL-6

Before treatment, there was no statistically significant difference in CRP levels between the test group (4.19 ± 4.16 mg/L) and the control group (4.00 ± 4.10 mg/L) (*p* > 0.05). However, after 10 days of treatment, the test group exhibited a greater decrease in CRP levels compared to the control group (−0.13 ± 2.85 mg/L vs*.* 0.25 ± 2.09 mg/L, *p* < 0.05). The IL-6 levels before treatment were 2.21 ± 3.77 pg/mL and 1.90 ± 3.43 pg/mL in the test and control groups, respectively, with no statistically significant difference (*p* > 0.05). After 10 days of treatment, there was no statistically significant difference in the IL-6 level-decreasing effect of both groups (*p* > 0.05) (Table 8).

**TABLE 8:** Changes in CRP and IL-6 levels^*^.

Category	Test group		Control group		*p*-values
N (Missing)	354 (2)		116 (1)		
**CRP**	MV	CV	MV	CV	
V1 (Baseline)	4.19 ± 4.16		4.00 ± 4.10		0.594
V5 (Day 10)	4.06 ± 4.29	−0.13 ± 2.85	4.25 ± 4.11	0.25 ± 2.09	0.033
N (Missing)	354 (2)		116 (0)^△^		
**IL-6**	MV	CV	MV	CV	
V1 (Baseline)	2.21 ± 3.77		1.90 ± 3.43		0.923
V5 (Day 10)	2.05 ± 3.59	−0.18 ± 3.68	1.74 ± 2.00	−0.15 ± 2.89	0.095

Statistics: *p*-values were calculated with Wilcoxon–Mann–Whitney test. Differences at baseline were compared based on measured values (MV) in both groups, while differences between both groups at different follow-up time points were compared based on change values (CV).

^*^CRP, values are in milligrams per liter (mg/L); IL-6, values are in picograms per milliliter (pg/mL).

^△^ The statistical analysis was performed after excluding one case of apparently abnormal extreme IL-6, values in the control group.

### 3.4 Safety assessment

Out of the total number of subjects in the test group, 16 (4.5%) experienced adverse events. Among these, 15 cases were classified as mild, and 1 was considered moderate. One of the mild adverse events, specifically abnormal liver function, was determined to have a potential relationship with the test drug and was classified as an adverse drug reaction. The remaining adverse events were deemed possibly unrelated to the test drug. At the end of the trial, 3 cases of mild adverse events were of unknown cause; 1 case of moderate adverse event was relieved; and the remaining 12 cases of mild adverse events were cured. A total of 3 cases (2.6%) of adverse events occurred in the control group, encompassing 2 mild adverse events and 1 moderate adverse event, all of which were determined to be possibly unrelated to the control drug. At the end of the trial, all adverse events were cured except for one mild adverse event of unknown cause (Table 9).

**TABLE 9:** Occurrence of adverse events (AE), adverse drug reaction (ADR), and serious adverse events (SAE).

Category	Test group (N = 356)	Control group (N = 117)	Total (N = 473)	*p*-values
**AE**	16 (4.5)	3 (2.6)	19 (4.0)	0.356
**SAE**	0 (0.0)	0 (0.0)	0 (0.0)	-
**ADR**	1 (0.3)	0 (0.0)	1 (0.2)	1.000
**AE unrelated to drug**	15 (4.2)	3 (2.6)	18 (3.8)	1.000
**AE severity**				0.298
mild	15 (4.2)	2 (1.7)	17 (3.6)	
moderate	1 (0.3)	1 (0.9)	2 (0.4)	
serious	0 (0.0)	0 (0.0)	0 (0.0)	
**AE results**				1.000
cured	12 (3.4)	2 (1.7)	14 (3.0)	
relieved	1 (0.3)	0 (0.0)	1 (0.2)	
unknown	3 (0.8)	1 (0.9)	4 (0.8)	
**Withdrew due to AE**	0 (0.0)	0 (0.0)	0 (0.0)	-
**Withdrew due to ADR**	0 (0.0)	0 (0.0)	0 (0.0)	-

Statistics: *p*-values were calculated with chi-square test or Fisher’s exact test.

In summary, the incidence of adverse events and adverse reactions (abnormal liver function) was 4.5% and 0.3%, respectively, in the test group after 10 days of treatment; in the control group, the incidence of adverse events was 2.6% and no adverse reactions occurred. The two groups had no statistical difference in the incidence of adverse events and adverse reactions (*p* > 0.05). The investigators concluded that all adverse events were unrelated to the trial drug except for one case of abnormal liver function. In addition, there were no serious adverse events in either group, and adverse events did not cause the withdrawal of any subject from the trial.

## 4 Discussion

The frequency of outdoor activities or physical exercise has increased with the growing emphasis on healthy lifestyles. However, this has led to an increased risk of injury, with acute soft tissue injuries being the most common (Sloan, 2008; Bleakley, 2013). Acute soft tissue injuries include sprains, contusions, falls, or impact injuries. An injury may result when the mechanical load on a particular tissue exceeds the tensile strength of the tissue. Local tissue trauma caused by such injury can lead to microcirculatory disturbance and an aseptic inflammatory response, resulting in local tissue pain, swelling, skin petechiae and ecchymosis, and even local functional limitations (Bleakley and Davison, 2010; Jones et al., 2020; Wang, 2021). Acute soft tissue injuries can adversely affect the work and life of a patient and often reduce productivity. Rapid and appropriate medical intervention for acute soft tissue injuries is essential for a favorable prognosis (Bleakley et al., 2010). Given that the P.R.I.C.E. or P.O.L.I.C.E. principles are not universal for all acute soft tissue injuries and the implementation is cumbersome and time-consuming, individuals prefer simple and effective treatment when the injury is mild. In addition, pain is the most common clinical manifestation of acute soft tissue injuries and often necessitates the use of oral analgesics. However, treatment with NSAIDs is limited because of the potential for adverse effects such as gastrointestinal irritation (Jones et al., 2020; Wang, 2021). Therefore, exploring new treatment options for acute soft tissue injuries is necessary.

TCM believes that acute soft tissue injuries can lead to the development of Qi stagnation and blood stasis syndrome, in which blood stasis predominates (Liu et al., 2012). The Golden Mirror of Medicine (edited by Wu, Q. in 1742) states: “The syndrome of traumatic injuries are treated based on the theory of blood; The symptoms of injury, which appear as swelling and pain, are caused by the clotting of blood stasis.” TCM theory holds that “obstruction causes pain.” After acute soft tissue injury, since blood stasis blocks the injured area and the movement of Qi is obstructed, typical localized stabbing pain occurs. This classical pain is key in diagnosing Qi stagnation and blood stasis syndrome. Additionally, if blood stasis does not disperse and overflows from the vessel to the muscle surface, swelling or petechiae may be observed, and these are also symptoms of Qi stagnation and blood stasis syndrome. Moreover, TCM considers a person as a whole. The pathological status of local Qi and blood may impact the Qi and blood status of various parts of the body, such as changes in tongue color (turning from normal light red into purple or dark) and pulse condition (diminished sense of pulse beat), and indirectly reflect Qi stagnation and blood stasis syndrome. Therefore, the main TCM principle for acute soft tissue injuries is to promote blood circulation and remove stasis (Liu et al., 2012; Wang et al., 2022).

Oral Chinese patent medicines (CPMs) are commonly used in TCM as a treatment method for acute soft tissue injuries because of their convenience of administration and lasting therapeutic effect, but solid CPMs are limited by their slow absorption rate (Wang, 2021). WHOL is a liquid CPM improved from a solid preparation. Liquid preparations have high bioavailability and are not associated with dysphagia (Bende et al., 2016; Liu et al., 2023). The development of WHOL provides a safe, efficient, convenient, and universal treatment for acute soft tissue injuries. This study was conducted to verify the clinical application of WHOL in the treatment of acute soft tissue injuries, obtain scientific and objective clinical data, and provide a clinical basis for the protection of Chinese medicine varieties.

WHOL is made up of five botanical drugs and functions to promote blood circulation, alleviate blood stasis, reduce swelling, and relieve pain. This preparation is primarily used for the treatment of acute soft tissue injury associated with qi stagnation and blood stasis syndrome caused by traumatic injuries and sprains. Acute and long-term toxicity tests showed no abnormal changes in various physiological indices of animals, indicating that the preparation was safe (see Supplementary Material for details). Modern research has found that the microcirculatory disturbance experienced by patients with blood stasis syndrome is associated with abnormal hemorheology, while the pain and swelling are caused by the release of various inflammatory mediators triggered by an aseptic inflammatory response following injury (Liu et al., 2012; Moshiri et al., 2017). Moreover, Pharmacodynamic studies have demonstrated that the metabolites in Angelica sinensis radix and Carthami flos significantly improve hemorheology in rats with blood stasis. Furthermore, combining these two botanical drugs enhances the hemorheological effects (Li et al., 2009; Liu et al., 2011; Yuan et al., 2019). The metabolites found in Saposhnikoviae radix, Arisaematis rhizoma, and Angelicae dahuricae radix have demonstrated notable anti-inflammatory and analgesic effects (Yang et al., 2020; Jin et al., 2021; Qi et al., 2021). In addition, WHOL has been proven to possess anti-inflammatory, analgesic, and hemorheological improvement properties (Deng et al., 2000). These findings provide a pharmacological basis for the use of WHOL in treating acute soft tissue injuries.

According to the relevant provisions of the Guiding Principles for the Protection of Varieties of Chinese Medicine (National Medical Products Administration, 2009), an application for the protection of varieties of TCM can be submitted if the effectiveness of the main treatment option is superior to that of similar varieties. The basic condition is to conduct clinical studies to prove that it has significant clinical advantages or superior efficacy compared to similar varieties. The study must have a sufficient sample size (not less than 300 subjects in the test group) and follow standardized adverse event evaluation procedures.

FFSTC is effective in treating acute soft tissue injuries (Zheng et al., 2012; Du, 2016; Ge and Fan, 2017; Chang et al., 2021). Notably, FFSTC demonstrated superior efficacy compared to similar Chinese medicines that relieve pain and swelling during a large-scale clinical trial (Zheng et al., 2012). Additionally, FFSTC demonstrated an analgesic effect equivalent to NSAIDs but better swelling reduction within 48 h (Ge and Fan, 2017). This study compared the efficacy of WHOL to that of FFSTC. Meanwhile, the sample size of the WHOL group exceeded 300 subjects (356 subjects). FAS was used in the efficacy analysis to better reflect the actual efficacy of the drug in clinical use, and SS was used in the adverse event assessment to ensure its reliability and comprehensiveness (JA, 1999).

WHOL outperformed FFSTC in various aspects. After 4 days of treatment, WHOL demonstrated superior efficacy in relieving pain at rest and on activity at the injury site. After 7 days, it demonstrated better effectiveness in alleviating pain on activity and reducing swelling at the injury site. Furthermore, after 10 days of treatment, WHOL was more effective in reducing swelling, improving the syndrome of qi stagnation and blood stasis, and lowering CRP levels compared to FFSTC. These findings highlight the potential clinical advantages of WHOL in managing acute soft tissue injuries. The disappearance time of pain at rest was 8 days in both groups and 9 days on activity in both groups. Meanwhile, medication compliance was satisfactory in both groups. In addition, there was no statistical difference in the incidence of adverse events and adverse reactions between the two groups during drug treatment. No serious adverse events occurred in either group, and no subjects were withdrawn because of adverse events. WHOL exhibited superior efficacy in relieving pain and swelling of acute soft tissue injury compared to the FFSTC. It also improved qi stagnation and blood stasis syndrome with fewer adverse effects and satisfactory compliance. These findings support the potential eligibility of WHOL for protection as a variety of TCM.

This study has some limitations. Quality of life was not assessed, and this limited the evaluation of the medication’s impact on improving quality of life. Additionally, a larger sample size is recommended for future clinical studies to further validate the efficacy and adverse effects of WHOL.

## 5 Conclusion

This study confirmed WHOL’s efficacy and safety in treating acute soft tissue injury associated with qi stagnation and blood stasis syndrome. It is particularly effective in relieving pain and swelling, with satisfactory patient compliance. Thus, WHOL is a safe and effective new alternative for patients with acute soft tissue injury associated with qi stagnation and blood stasis syndrome in the future.

## Data Availability

The original contributions presented in the study are included in the article/Supplementary Material, further inquiries can be directed to the corresponding authors.
