# Supplementary material for: Efficacy and safety of Wuhu oral liquid in treating acute soft tissue injuries: a multicenter, randomized, double-blind, double-dummy, parallel-controlled trial
Source: Front Pharmacol. 2024 Feb 23;15:1335182. doi: 10.3389/fphar.2024.1335182 (PMC10921885; doi:10.3389/fphar.2024.1335182)
Supplement: Supplementary file 1 [file Table1.DOCX]

***Supplementary Material***

**1. The fingerprint of Wuhu Oral Liquid**

**2. Quality Control in the Preparation of Wuhu Oral Liquid**

**3. Optimal Preparation Method for Wuhu Oral Liquid**

**4. Stability Study of Wuhu Oral Liquid**

**5. Pharmacological Study of Wuhu Oral Liquid**

**6. Toxicological Study of Wuhu Oral Liquid**

**1 The fingerprint of Wuhu Oral Liquid**

Method: Chromatographic column: Platisil ODS C18 (4.6×250 mm,5μm); mobile phase: gradient elution with acetonitrile-0.1% formic acid water as mobile phase (Table 1); Flow rate: 0.6 mL/min； Sample volume: 10μL; Detection Wavelength: 280 nm; Column temperature: Room temperature.

**Table 1.** Acetonitrile-0.1% formic acid water gradient elution conditions

| Time/min | Acetonitrile/% | 0.1%formic acid water/% |
| --- | --- | --- |
| 0 | 5 | 95 |
| 65 | 42 | 58 |
| 70 | 5 | 95 |

Steps:

**1.1 Preparation of Test Solution**

Precisely measure 5 mL of Wuhu Oral Liquid sample into a conical flask, add 25 mL of methanol, weigh, ultrasonic (power 180 W, frequency 40 KHz) for 15 min, cool down, make up the lost weight with methanol, shake well, filtered with 0.22 μm microporous membrane, and take the filtrate as the test solution.

**1.2 Preparation of Control Solution**

Take botanical drugs including Angelica sinensis radix, Carthami flos, Saposhnikoviae radix, Arisaematis rhizoma and Angelicae dahuricae radix as control drugs. Prepare control drug samples according to the preparation process of Wuhu Oral Liquid, respectively. Take 5 mL of each control drug sample and prepare control drug solutions according to the method used for the preparation of the test solution.

**1.3 Preparation of negative sample solution**

Take Wuhu oral liquid lacking Chinese Angelica, lacking safflower, lacking Saposhnikovia divaricata, lacking Dahurian Angelica, and lacking prepared Rhizoma Arisaematis, respectively. Prepare single negative sample solutions according to the method used for the preparation of the test solution.

Results: A total of 26 common peaks that can be used as fingerprint peaks for Wuhu oral liquid were finally identified, generating a control fingerprint (Figure 1).


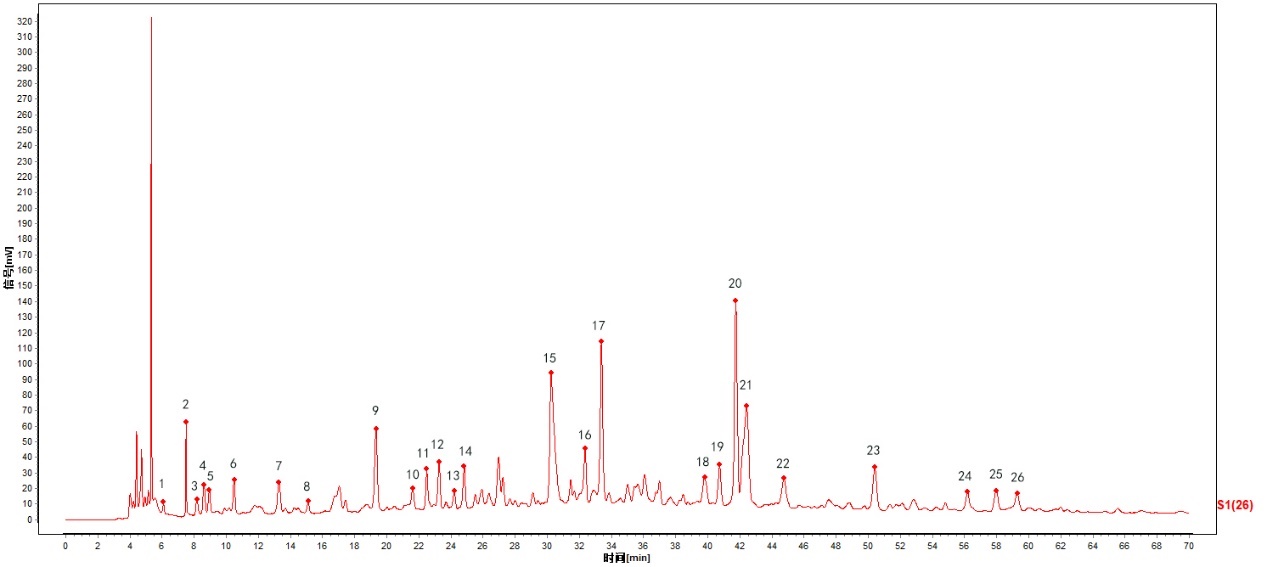


**Figure 1.** HPLC Fingerprint of Wuhu oral liquid (Control)

Import the chromatograms of 10 batches of Wuhu oral liquid into the "Chinese Medicine Chromatographic Fingerprint Similarity Evaluation System Software (2012 Edition)," with S1 set as the reference fingerprint. Use the median method to generate test fingerprints and the control fingerprint, perform peak matching, select chromatographic peaks with distinct features and large peak areas as common peaks, and finally, a total of 26 common peaks were identified as fingerprints for Wuhu oral liquid (Figure 2). Among them, peak 15 showed good stability and a large peak area, identified as hydroxysafflor yellow A, serving as a reference peak.


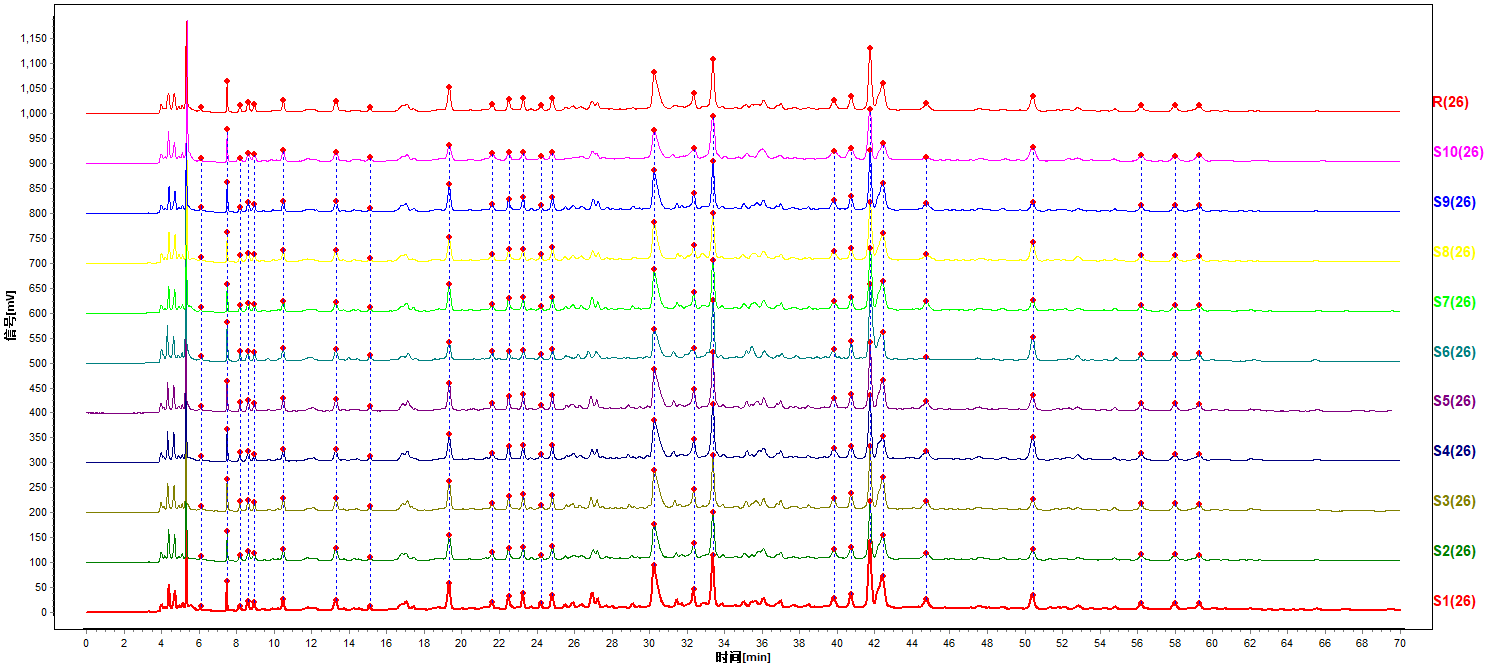


**Figure 2.** HPLC Fingerprint of 10 batches of Wuhu oral liquid

Through comparative analysis, among the 26 common peaks in the fingerprint, 5 originated from Angelica sinensis radix, 11 from Carthami flos, 5 from Saposhnikoviae radix, and 2 from Angelicae dahuricae radix, with no chromatographic peaks originating from Arisaematis rhizoma. 1st, 2nd, and 10th peaks had retention times and spectral patterns inconsistent with the five botanical drugs, and these three chromatographic peaks were present in the negative control, suggesting that they may be new compounds generated during the solution preparation process and were not assigned. The results are shown in Table 2.

**Table. 2** The attribution of 26 common peaks

| Peak No. | Retention time（min） | Attribution | Peak No. | Retention time（min） | Attribution |
| --- | --- | --- | --- | --- | --- |
| 1 | 6.104 | / | 14 | 24.863 | Carthami flos |
| 2 | 7.537 | / | 15 | 30.126 | Carthami flos |
| 3 | 8.181 | Carthami flos | 16 | 32.405 | Carthami flos |
| 4 | 8.601 | Angelica sinensis radix | 17 | 33.456 | Saposhnikoviae radix |
| 5 | 8.911 | Saposhnikoviae radix | 18 | 39.804 | Carthami flos |
| 6 | 10.464 | Carthami flos | 19 | 40.827 | Saposhnikoviae radix |
| 7 | 13.295 | Saposhnikoviae radix | 20 | 41.862 | Saposhnikoviae radix |
| 8 | 15.119 | Carthami flos | 21 | 42.434 | Carthami flos |
| 9 | 19.352 | Angelica sinensis radix | 22 | 44.699 | Angelica sinensis radix |
| 10 | 21.706 | / | 23 | 50.498 | Angelica sinensis radix |
| 11 | 22.523 | Carthami flos | 24 | 56.311 | Carthami flos |
| 12 | 23.263 | Carthami flos | 25 | 58.089 | Angelicae dahuricae radix |
| 13 | 24.220 | Angelica sinensis radix | 26 | 59.399 | Angelicae dahuricae radix |

"/" indicates that no attribution has been provided.


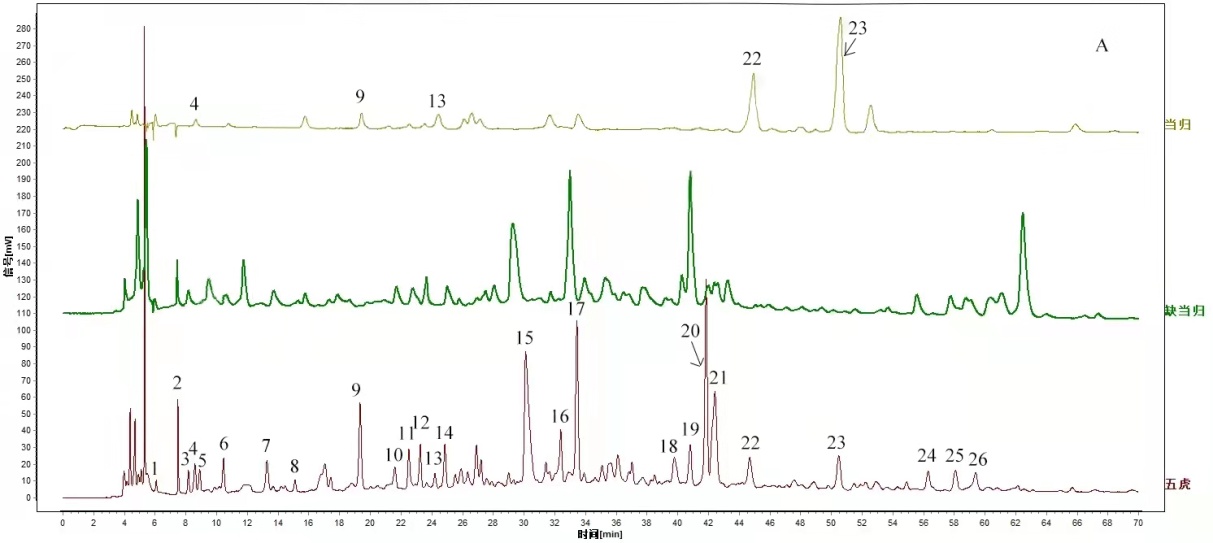


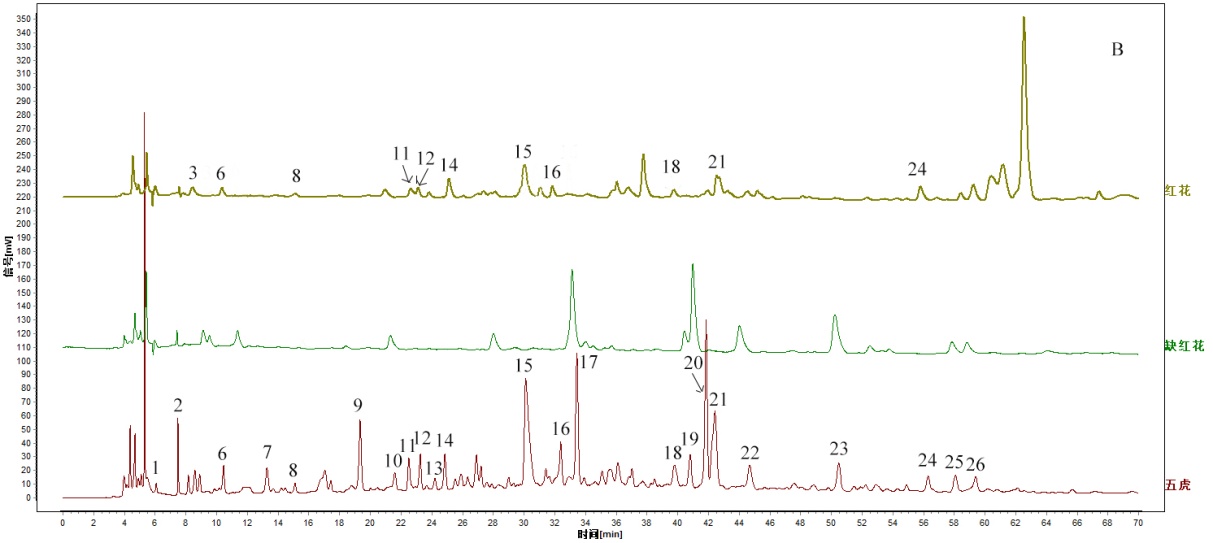


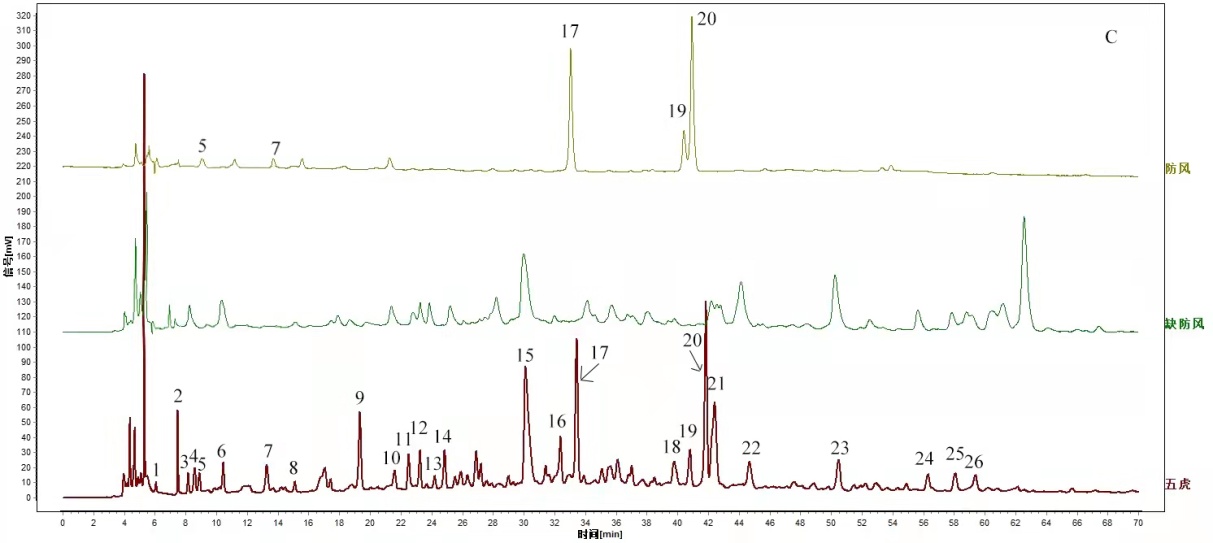


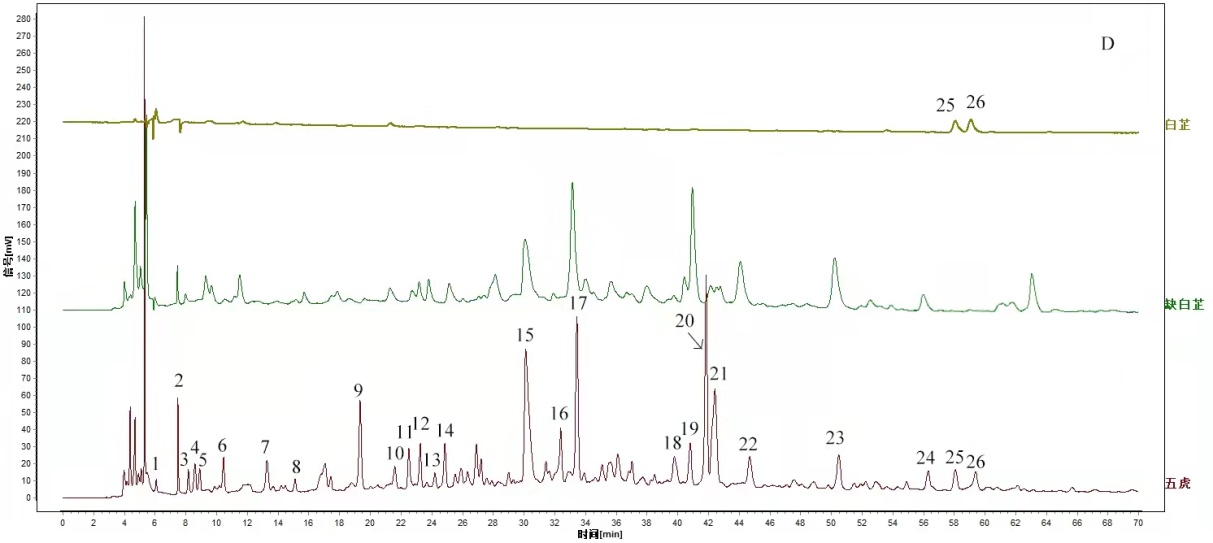


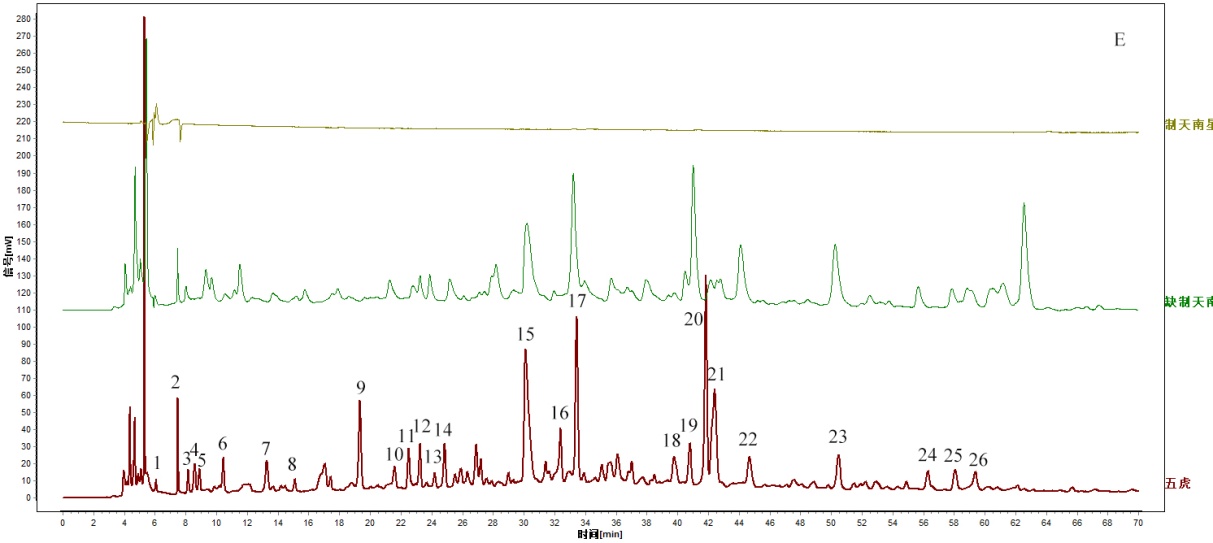


Note：3 fingerprints per image.Example with A image (from top to bottom):Angelica sinensis radix, Wuhu oral liquid lacks Angelica sinensis radix; Wuhu oral liquid.

（A：Angelica sinensis radix；B：Carthami flos；C：Saposhnikoviae radix；D：Angelicae dahuricae radix；E：Arisaematis rhizoma）

**Figure 3.** Chromatogram of botanical drugs and Wuhu oral liquid (A-E)

This section established a fingerprint detection method for Wuhu oral liquid using high-performance liquid chromatography. The chromatographic conditions were optimized by examining the detection wavelength, chromatographic column, mobile phase, mobile phase gradient, and flow rate. The sample solution preparation method was determined by investigating preparation methods, preparation solvents, processing time, liquid-to-material ratio, etc. Simultaneously, methodological investigations such as instrument precision, repeatability, and stability were conducted, yielding satisfactory results. Fingerprinting was established through the analysis of 10 batches of Wuhu oral liquid samples, identifying a total of 26 common peaks, 5 originating from Angelica sinensis radix, 11 from Carthami flos, 5 from Saposhnikoviae radix, and 2 from Angelicae dahuricae radix, with no chromatographic peaks originating from Arisaematis rhizoma. 1st, 2nd, and 10th Peaks had retention times and spectral patterns inconsistent with the five herbal flavors, and these three chromatographic peaks were present in the negative control, suggesting that they may be new compounds generated during the solution preparation process and were not assigned. **Similarity evaluation was conducted on 10 batches of Wuhu oral liquid samples, and the similarity coefficients were all greater than 0.9, indicating good consistency in quality.**

**2 Quality Control in the Preparation of Wuhu Oral Liquid**

Ensuring the stability of ferulic acid content is a crucial aspect of quality control in the preparation of the Wuhu oral liquid. The method employs high-performance liquid chromatography with the following specifications: C18 column (125×4mm, 5μm); mobile phase: methanol-1% acetic acid (24:76); theoretical plate number not less than 1500, calculated for ferulic acid; detection wavelength: 313nm. The results indicate a linear range for ferulic acid content of 0.11 to 0.385μg, with a correlation coefficient (r) of 0.9999. The average recovery rate is 100.79%, and the relative standard deviation (RSD) is 1.87%. This method is characterized by its simplicity, reliability, ease of operation, sensitivity, accuracy, specificity, and excellent reproducibility. It is suitable for determining ferulic acid content in Wuhu oral liquid and ensuring its quality control. **The approved standard for ferulic acid content in every 1ml of Wuhu oral liquid is not less than 0.05mg.**

**Detailed steps are as follows:**

**2.1 Instruments and Reagents**

High-performance Liquid Chromatography System: HP SERIES 1100; Reference substance: Ferulic Acid(110773-200015,99.4%) was purchased from the China National Institutes for Food and Drug Control; Methanol, Ethyl acetate (chromatography pure, sourced from Huanyan Chemical Factory, Zhejiang); Hcl, Glacial acetic acid (analytically pure, obtained from Shantou Xinning Chemical Factory).

**2.2 Chromatographic Conditions**

Column: HP Hypersil C18 (125×4mm, 5μm); Mobile phase: Methanol-1% acetic acid (24:76); Flow rate: 1.0ml/min; Column temperature: Room temperature; Detection Wavelength: 313nm. Under these conditions, ferulic acid and other impurity peaks achieve baseline separation, with minimal absorption observed in the negative liquid at 11 minutes. This makes the method suitable for quantitative analysis. The theoretical plate number, calculated for ferulic acid, is not less than 1500.


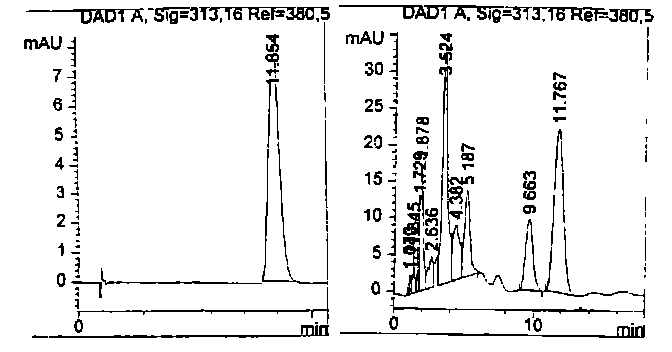


**Figure 4.**　Standard product chromatogram　　**Figure 5.**　sample chromatogram

**2.3 Preparation of Test Solution**

Precisely measure 15ml of the sample, place it in a separating funnel, adjust the pH to 2-3 with dilute HCl, and extract with ethyl acetate five times (15ml, 15ml, 10ml, 10ml, 10ml). Combine the extracts, evaporate in a water bath, dissolve in 50% methanol, transfer to a 25ml volumetric flask, and dilute with 50% methanol to the mark.

**2.4 Standard Curve**

Precisely measure 1ml, 1.5ml, 2ml, 2.5ml, 3ml, and 3.5ml of the ferulic acid reference solution (0.22mg/ml) in 50% methanol. Place each in separate 10ml volumetric flasks, dilute with 50% methanol to the mark, inject 5μl of each into the HPLC system, measure the peak area, perform linear regression with concentration (μg/ml) against peak area, and obtain the standard curve equation: Y = 4337.5X - 10.09096; r = 0.9999. The results indicate a robust linear relationship for ferulic acid within the concentration range of 0.110 to 0.385μg/ml.

**2.5 Precision Test**

Conduct five consecutive injections of the same sample, ensuring that the RSD of the peak area is below 2%. The details are shown in Table 3.

**Table. 3** Precision Test

| Frequency | 1 | 2 | 3 | 4 | 5 | RSD |
| --- | --- | --- | --- | --- | --- | --- |
| Peak area | 708.6 | 709.2 | 711.3 | 710.1 | 711.8 | 0.19% |

**2.6 Stability Test**

For the identical sample, perform injections at 30-minute intervals over a 2.5-hour period, with the requirement that the relative standard deviation of the peak area remains below 2%. The details are shown in Table 4.

**Table. 4** Stability Test

| Time(min) | 0 | 30 | 60 | 90 | 120 | 150 | RSD |
| --- | --- | --- | --- | --- | --- | --- | --- |
| Peak area | 708.6 | 711.3 | 711.8 | 712.4 | 712.1 | 713.0 | 0.217% |

**2.7 Reproducibility Test**

Extract five portions from the same batch of samples, follow the preparation procedure outlined in Section 2.3, inject, and observe a resulting RSD% of 2.64% (n=5).

**2.8 Recovery Rate Test**

Accurately draw 10ml of the sample solution, add the standard solution of ferulic acid following the procedure in Section 2.3, inject, and determine an average recovery rate of 100.9%, with an RSD of 1.87% (n=5).

**2.9 Sample Determination**

Process ten sample batches according to the procedure in Section 2.3 and inject. Verify that the ferulic acid content in all samples consistently exceeds 0.05mg/ml. The details are shown in Table 5.

**Table. 5** Sample Determination

| Batch number | Ferulic acid content | Batch number | Ferulic acid content |
| --- | --- | --- | --- |
| 970303 | 0.0592 | 970413 | 0.0554 |
| 970304 | 0.0562 | 970515 | 0.0551 |
| 970305 | 0.0541 | 970516 | 0.0570 |
| 970411 | 0.0537 | 970517 | 0.0563 |
| 970412 | 0.0558 | 970518 | 0.0573 |

**3 Optimal Preparation Method for Wuhu Oral Liquid**

The optimal drug ratios and steps for preparing the Wuhu oral liquid described in this study are as follows. The following preparation method ensured the stability of the chemical compound ferulic acid in Wuhu oral liquid.

The raw materials for making 1000 mL of Wuhu Oral Liquid include Angelica sinensis (128 g), Flos Carthami (128 g), Radix Saposhnikoviae (128 g), processed Rhizoma Arisaematis (128 g), Angelica dahurica (88 g), Tween-80 (3 g), steviol glycosides (1 g), sodium metabisulfite (1 g), and calcium disodium edetate (2 g). Angelica sinensis, Flos Carthami, Radix Saposhnikoviae, Rhizoma Arisaematis (processed), Angelica dahurica were mixed with 8 times their weight of 70% ethanol. The mixture underwent reflux extraction 3 times, with each extraction lasting 2 hours. After filtration, the filtrate was combined, and the ethanol was recovered. The combined filtrate was then concentrated under reduced pressure to a relative density of 1.07 (50°C) and stored at room temperature. A total of 60mL of 2% chitosan-1% acetic acid solution was added to the obtained concentrated solution and stirred thoroughly, heat to 60℃ for 10 min before cooling to room temperature. The solution was then filtered to obtain a clear solution. A total of 167 mL of edible alcohol (60%) was added to the clear solution and stirred thoroughly. Next, diatomite (1% w/v) was added with continuous stirring of the solution. The solution was then filtered using a plate and frame filter, and the filtrate was collected. Tween-80, steviol glycosides, sodium metabisulfite, and calcium disodium edetate were then added to the mixture according to the recipe and thoroughly stirred. Purified water was then added to make up a volume of 1000 mL. The pH was then adjusted to 5.0 before filtration.

After the solution was prepared, it was filled into containers, with each filling containing 10 mL. The filled containers are then subjected to sterilization at 100°C for approximately 30 minutes. The reagents or instruments used were all conventional products available through formal channels. Among them, a chitosan clarifying agent was provided by Zhejiang Golden Shell Pharmaceutical Co. Ltd.

**The proportion of botanical drugs contained in each standard dose (10ml) of WHOL is as follows:** *Angelica sinensis* (Oliv.) Diels [Apiaceae; Angelica sinensis radix]: 1.28g (equally 1.28 g botanical drug, same as below), *Carthamus tinctorius* L. [Asteraceae; Carthami flos]: 1.28g, *Saposhnikovia divaricata* (Turcz. ex Ledeb.) Schischk. [Apiaceae; Saposhnikoviae radix]: 1.28g, *Arisaema erubescens* (Wall.) Schott [Araceae; Arisaematis rhizoma]: 1.28g; *Angelica dahurica* (Hoffm.) Benth. & Hook.f. ex Franch. & Sav*.* [Apiaceae; Angelicae dahuricae radix]:0.88g.**This product is a brown liquid; sweet, slightly bitter taste. Expiration date:18 months.** The recommended dose of Wuhu Oral Liquid prepared using the described method is 10mL twice daily.

The China National Intellectual Property Administration (CNIPA) granted approval for the invention patent (Patent No. ZL201610113641.7) for the preparation method of Wuhu Oral Liquid. This patented method offers significant advantages, including improved utilization of pharmaceutical raw materials, enhanced efficacy and safety of the drug, and improved stability of the drug.

**4 Stability Study of Wuhu Oral Liquid**

Place samples of Wuhu oral liquid (prepared according to the method mentioned above) under accelerated conditions at a high temperature (60℃). Following the method (Part 2 above), assess the content of ferulic acid in Wuhu oral liquid samples on days 0, 5, and 10. After ten days under high-temperature conditions, observe a decrease in content of less than 5% while maintaining levels above the minimum standard for ferulic acid. This indicates excellent high-temperature stability for Wuhu oral liquid, as outlined in Table 6.

**Table 6.** Results of determining ferulic acid content in Wuhu Oral Liquid samples under high temperature (60℃) (Unit: mg/mL)

| **Duration of exposure** | **0 days** | **5 days** | **10 days** | **Decrease in 10 days (%)** |
| --- | --- | --- | --- | --- |
| **Ferulic acid content** | **0.0753** | **0.0736** | **0.0722** | **4.2%** |

Furthermore, conduct a long-term stability experiment on Wuhu oral liquid samples (24 months). Assess the content of ferulic acid in Wuhu oral liquid samples on days 0, 3, 6, 12, 18, and 24. Throughout the 24-month period, note a decrease in content of less than 5% while maintaining levels above the minimum standard for ferulic acid. This affirms outstanding long-term stability for Wuhu oral liquid, as depicted in Table 7.

| **Months** | **0** | **3** | **6** | **12** | **18** | **24** | **Decrease in 24 Months (%)** |
| --- | --- | --- | --- | --- | --- | --- | --- |
| **Ferulic acid content** | **0.071** | **0.071** | **0.070** | **0.070** | **0.069** | **0.068** | **4.3%** |

**Table 7.** Results of determining ferulic acid content in Wuhu oral liquid samples under long-time preservation (Unit: mg/mL)

**5 Pharmacological Study of Wuhu Oral Liquid**

Experimental Materials: Wuhu Oral Liquid (prepared using the method mentioned above, batch number:20131104), Wuhu Powder (manufactured by Beijing Tongrentang Pharmaceutical Co., Ltd., batch number: 20130812), prepared to the required concentration with a 10% ethanol solution. The control group received an equivalent volume of a 10% ethanol solution. Aspirin, produced by Shandong Xinhua Pharmaceutical Co., Ltd., batch number: 20130402. Pethidine hydrochloride(PH), manufactured by Yichang Renfu Pharmaceutical Co., Ltd., batch number: 20130303.

Animals: Kunming mice, 18-22g, with an equal distribution of males and females(provided by Beijing HFK Biotechnology Co., Ltd).

**5.1 Analgesic Effect (Acetic Acid Writhing Test in Mice)**

Mice were randomly divided into four groups and administered either 10% ethanol or the drug via gavage. After one hour, 0.1ml/10g of 0.6% acetic acid solution was injected intraperitoneally, and the number of writhing movements within 20 minutes was observed. The results indicate that Wuhu Oral Liquid reduces the number of writhing movements in mice significantly compared to the control group. See Table 8 for detailed results.

**Table 8.** Determination of the analgesic effect of Wuhu Oral Liquid using the acetic acid writhing test**（±SD）**

| Group | Dose (g/kg) | Number | Writhing times |
| --- | --- | --- | --- |
| Control group | 0 | 10 | 28.8±4.57 |
| PH group | 0.05 | 10 | 11.9±3.46^#^ |
| Wuhu Power group | 6 | 10 | 19.9±3.51^#^ |
| Wuhu Oral Liquid Group | 6 | 10 | 15.6±2.63^#^ |

Note: ^#^P<0.01 (vs. control group)

Furthermore, Wuhu Oral Liquid samples, after one year of storage, underwent the acetic acid writhing test in mice following the above method. The results demonstrate that even after one year of storage, the analgesic effect of the Wuhu Oral Liquid group did not show a significant decrease and remained statistically significant compared to the control group. See Table 9 for detailed results.

**Table 9.** Determination of the analgesic effect of Wuhu Oral Liquid after one year of storage using the acetic acid writhing Test **(±SD)**

| Group | Dose (g/kg) | Number | Writhing times |
| --- | --- | --- | --- |
| Control group | 0 | 10 | 27.5±3.62 |
| PH group | 0.05 | 10 | 12.1±3.59^#^ |
| Wuhu Power group | 6 | 10 | 19.6±3.72^#^ |
| Wuhu Oral Liquid Group | 6 | 10 | 15.9±2.97^#^ |

Note: ^#^P<0.01 (vs. control group)

**5.2 Anti-inflammatory Effect(Xylene-induced Mouse Ear Edema)**

Mice were randomly divided into five groups. 45 minutes before inducing inflammation, they were administered either 10% ethanol or the drug via gavage. 0.05ml of xylene was applied to the front and back of the right ear of each mouse, while the left ear served as a control. After 20 minutes, the mice were euthanized, and both ears were dissected along the ear outline. Slices of ears were punched out using an 8mm diameter punch at the same location, weighed, and calculated based on a 100g weight of the left ear. The percentage increase in weight due to inflammation in the right ear was then determined. The results indicate that Wuhu Oral Liquid can effectively inhibit xylene-induced inflammation in the mouse ear. See Table 10 for detailed results.

**Table 10.** Anti-inflammatory effects of Wuhu oral liquid (**±SD)**

| Group | Dose (g/kg) | Number | Increased ear inflammation% |
| --- | --- | --- | --- |
| Control group | 0 | 10 | 159.6±34.2 |
| Aspirin group | 0.05 | 10 | 95.97±39.5^*^ |
| Wuhu Power group | 6 | 10 | 105.3±45.7^*^ |
| Wuhu Oral Liquid Group | 6 | 10 | 99.5±58.2^*^ |

Note: ^*^P<0.01 (vs. control group)

Additionally, Wuhu Oral Liquid samples after one-year storage underwent the xylene-induced inflammation experiment following the above method. The results demonstrate that even after one year of storage, the anti-inflammatory efficacy of the Wuhu Oral Liquid group did not significantly decrease and remained statistically significant compared to the control group. See Table 11 for detailed results.

**Table 11.** Determination of the analgesic effect of Wuhu Oral Liquid after one year of storage (**±SD)**

| Group | Dose (g/kg) | Number | Increased ear inflammation% |
| --- | --- | --- | --- |
| Control group | 0 | 10 | 157.4±54.1 |
| Aspirin group | 0.05 | 10 | 96.35±42.8^*^ |
| Wuhu Power group | 6 | 10 | 104.6±41.4^*^ |
| Wuhu Oral Liquid Group | 6 | 10 | 100.3±46.3^*^ |

Note: ^*^P<0.01 (vs. control group)

**6 Toxicological Study of Wuhu Oral Liquid**

**6.1 Acute Toxicity**

Due to the low toxicity of this drug, the LD_50_ could not be determined; therefore, the Maximum Tolerated Dose (MTD) was assessed.

**Experimental Animals:** Kunming mice, weighing 18-22g, with an equal distribution of males and females, provided by the Animal Institute of the First Military Medical University, certificate number: 97A028

**Test drug:** Wuhu Oral Liquid extract, where each 1g of the extract is equivalent to 3g of the crude drug, provided by the Animal Institute of the First Military Medical University, Batch No. 970903.

**Experimental Method:** After a 3-day acclimatization in a room with consistent temperature (24-28℃), 20 mice were fasted for 12 hours before being orally gavaged with Wuhu Oral Liquid extract. Each time, 0.5ml/10g (maximum allowable volume) of the extract was administered orally, with the maximum allowable concentration in a 10% ethanol solution as the solvent. The mice were observed for signs of toxicity and survival for seven days.

**Experimental Results:** The experimental mice were orally gavaged with Wuhu Oral Liquid extract at the maximum allowable concentration and volume, resulting in a dose of up to 150g/kg (equivalent to 750 times the recommended clinical daily dose). All mice survived within seven days, with an increase in activity and redness of ears and tails observed 10 minutes after administration. Normal behavior, no arching of the back, and unchanged fur were observed 2 hours later. The mice showed an increase in body weight within the first seven days (Table 12). The MTD for orally gavaged mice with Wuhu Oral Liquid was determined to be >150g/kg (calculated based on the crude drug). The results indicate that oral gavage administration of Wuhu Oral Liquid to mice is essentially non-toxic.

**Conclusion:** The acute toxicity test, using the maximum gavaging concentration and maximum allowable volume, showed that all mice survived without toxic symptoms within seven days, with a natural increase in body weight. The MTD (calculated based on the crude drug) was determined to be 150g/kg.

**Table 12.** Changes in body weight in the acute toxicity test of Wuhu Oral Liquid in mice(**±SD, g**)

|  | Female mice **♀** | | Male mice ♂ | |
| --- | --- | --- | --- | --- |
| Time (days) | D0 | D7 | D0 | D7 |
|  | 19.1±1.1 | 25.9±1.3 | 19.5±1.1 | 26.2±1.7 |

**6.2 Long-term Toxicity Test**

**Drug:** Wuhu Oral Liquid Extract Concentrate, equivalent to 3g per ml of the crude drug, Batch No. 980608. It was thoroughly shaken before each use. The doses of Wuhu Oral Liquid used in this study were calculated based on the quantity of the crude drugs.

**Animals:** SD rats, seven weeks old, weighing 170-190g, with an equal distribution of males and females. These rats were provided by the Experimental Animal Center of the First Military Medical University, with the certificate number 97A033.

**Method:** Rats were housed in a room with consistent temperature (25±1℃) for one week. A total of 80 rats exhibiting normal behavior were selected, and male and female rats were individually numbered based on their weight. Subsequently, they were randomly divided into four groups, each consisting of 20 rats (10 males and 10 females). The high-dose group and low-dose group received daily oral doses of 30g/kg and 6g/kg, respectively (equivalent to 150 times and 30 times the recommended clinical dose), administered at a rate of 1ml per 100g of body weight. The control group received an equivalent volume of physiological saline, while the solvent group was administered an equivalent volume of 10% ethanol. The administration continued for four weeks, during which the rats' general behavior, fur condition, and feces were observed. Body weight was measured weekly, and after one month of cessation, a random selection of 5 rats from each group (male and female) underwent blood analysis (RBC, Hb, WBC and its classification, Platelets, and coagulation time) and 12 biochemical indicators (BUN, CR, GLU, CHOL, TBIL, TP, ALB, G, ALT, AST, ALP, γ-GT). Pathological examinations were conducted on the heart, liver, spleen, lungs, kidneys, brain, adrenal glands, thyroid, thymus, prostate, ovaries and uterus, testicles, and epididymis—14 organ systems in total. The weights of the aforementioned organs were measured, and the organ coefficients were calculated. The other half of the rats discontinued treatment and were observed for an additional two weeks before repeating the above experiments.

**Results:** (1) General Conditions: Rats in the drug group, solvent group, and control group exhibited normal activity, glossy fur closely adhering to the body, normal feces and diet, and continuous weight gain. There were no significant differences between the groups(*p*>0.05)(See Table 13 for detailed results). (2) Hematological Parameters: Hematological indicators, including RBC, Hb, WBC, and their classifications, Platelets, and coagulation time, were within the normal range for the drug group, solvent group, and control group. No significant differences were observed between the groups(*p*>0.05)(See Table 14 for detailed results). (3) Biochemical Parameters: Biochemical indicators, including BUN, CR, TP, AIB, G, AST, AIP, GLU, TBIL, and γ-GT, showed no significant differences among the drug groups, solvent group, and control group rats(*p*>0.05). However, there were significant differences in CHOL and ALT in the solvent group(See Table 15 for detailed results). (4) Pathological Examination: Relative organ weights (organ weight g/100g body weight) of major organs in rats of the drug group were not significantly different from those in the control group(*p*>0.05)(See Table 16 for detailed results). Results of pathological examination revealed mild fatty liver changes in the liver of one rat in the solvent group, two rats in the low-dose group, and three rats in the high-dose group. No abnormalities were detected in the organs of animals in other groups. No abnormalities were observed in the organs of rats half a month after discontinuation.

**Table 13.** Changes in the body weight of rats in the long-term toxicity test of Wuhu Oral Liquid (**±SD**)

| Time (days) | High-dose group | Low-dose group | Solvent group | Control group |
| --- | --- | --- | --- | --- |
| 0 | 189.8±14.8 | 185.8±14.7 | 185.5±113.2 | 190.5±10.3 |
| 7 | 211.8±21.1 | 210.5±20.0 | 216.2±18.3 | 220.6±20.7 |
| 14 | 234.2±28.3 | 235.5±25.7 | 242.8±25.5 | 248.0±25.6 |
| 21 | 257.5±30.3 | 260.0±26.2 | 267.2±33.8 | 270.1±30.3 |
| 28 | 276.3±34.6 | 269.8±28.5 | 278.5±37.2 | 287.9±36.0 |
| 35 | 288.5±40.0 | 290.0±35.6 | 293.0±39.8 | 300.0±33.2 |
| 42 | 293.6±42.7 | 302.0±39.7 | 304.0±42.4 | 311.0±37.8 |

Note: n=20, n=10 from day 35

**Table 14.** Effects of the long-term toxicity test of Wuhu Oral Liquid on blood routine in rats(**±SD**)

|  |  |  |  |  |  | Classification(%) | |  |
| --- | --- | --- | --- | --- | --- | --- | --- | --- |
| Group | Time(days) | RBC(×10^12^L) | Hb(g/L) | Plat(×10^4^/mm^3^) | WBC(×10^9^/L) | Lymphocyte | Neutrophil | BCT(s) |
| Control group | D28 | 9.01±0.58 | 14.8±1.1 | 75.4±13.2 | 13.0±3.3 | 78.0±3.9 | 19.5±4.4 | 116±30 |
|  | D42 | 9.21±0.62 | 15.0±1.2 | 76.5±15.4 | 14.3±3.6 | 77.7±4.2 | 20.0±4.6 | 120±40 |
| Solvent group | D28 | 8.80±0.48 | 14.5±1.0 | 84.4±13.6 | 12.5±3.1 | 79.9±4.0 | 19.2±4.1 | 114±28 |
|  | D42 | 9.30±0.48 | 15.0±1.3 | 73.4±13.2 | 15.5±3.5 | 78.2±4.2 | 19.9±4.0 | 132±37 |
| High-dose group | D28 | 8.49±0.85 | 14.1±1.4 | 77.0±16.2 | 12.7±3.8 | 78.6±5.1 | 20.4±4.7 | 121±46 |
|  | D42 | 8.99±0.72 | 14.7±1.2 | 76.1±15.0 | 14.7±3.7 | 77.4±4.6 | 21.0±5.2 | 125±46 |
| Low-dose group | D28 | 9.01±0.47 | 14.6±1.0 | 84.2±14.3 | 11.7±2.6 | 79.9±3.7 | 18,8±4.0 | 140±45 |
|  | D42 | 9.37±0.79 | 15.1±1.1 | 78.8±12.7 | 15.0±4.1 | 78.9±3.9 | 19.2±4.4 | 125±45 |

Note: n=10

**Table 15.** Effects of the long-term toxicity test of Wuhu Oral Liquid on biochemical parameter in rats(**±SD**)

| Indicator | Time (days) | Control group | Solvent group | High-dose group | Low-dose group |
| --- | --- | --- | --- | --- | --- |
| BUN(mmol/L) | D28 | 7.61±0.64 | 6.74±1.21 | 7.65±1.53 | 7.85±1.17 |
|  | D42 | 6.73±0.94 | 7.61±0.64 | 6.77±1.09 | 7.46±1.11 |
| CR(μmol/L) | D28 | 67.2±5.5 | 61.8±6.3 | 65.7±7.8 | 64.9±5.7 |
|  | D42 | 63.4±6.8 | 67.2±5.51 | 62.5±7.4 | 66.7±12.5 |
| GLU(μmol/L) | D28 | 2.82±0.62 | 2.97±0.77 | 3.15±0.59 | 3.32±0.52 |
|  | D42 | 2.61±0.38 | 2.82±0.62 | 2.76±0.62 | 2.57±0.35 |
| CHOL(μmol/L) | D28 | 1.62±0.24 | 1.65±0.25 | 1.77±0.14 | 1.55±0.28 |
|  | D42 | 1.57±0.28 | 1.70±0.27 | 1.49±0.34 | 1.57±0.19 |
| TBIL(μmol/L) | D28 | 4.09±2.27 | 3.23±1.39 | 3.07±3.32 | 2.96±1.55 |
|  | D42 | 4.11±3.10 | 4.08±2.24 | 4.03±2.73 | 4.17±3.66 |
| TP(g/L) | D28 | 76.5±5.50 | 79.0±4.80 | 76.9±3.11 | 81.9±5.70 |
|  | D42 | 74.2±4.37 | 76.5±5.48 | 75.0±4.74 | 73.1±5.99 |
| ALB(g/L) | D28 | 34.1±3.00 | 36.9±3.00 | 36.2±2.04 | 37.6±2.01 |
|  | D42 | 33.7±1.89 | 35.3±3.89 | 34.8±3.08 | 34.8±2.78 |
| G(g/L) | D28 | 42.3±4.60 | 42.1±4.30 | 40.7±3.40 | 44.3±4.20 |
|  | D42 | 40.8±4.52 | 41.2±4.32 | 40.2±3.55 | 37.3±7.02 |
| ALT(μ/L) | D28 | 47.5±10.9 | 42.3±7.4 | 54.5±11.9 | 51.1±13.8 |
|  | D42 | 49.6±9.62 | 47.5±10.86 | 50.5±8.29 | 52.0±12.9 |
| AST(μ/L) | D28 | 231.2±26.1 | 212.9±36.2 | 228.5±31.8 | 229.8±32.7 |
|  | D42 | 240.1±30.1 | 231.2±26.25 | 221.3±19.79 | 243.6±46.31 |
| ALP(μ/L) | D28 | 131.6±40.7 | 129.8±30.8 | 129.6±56.7 | 130.5±57.1 |
|  | D42 | / | / | / | / |
| r-GT(μ/L) | D28 | 1.20±0.41 | 1.5±0.53 | 1.3±0.50 | 1.4±0.52 |
|  | D42 | 1.30±0.48 | 1.10±0.32 | 1.2±0.42 | 1.2±0.42 |

Note: n=10; abbreviation: Blood Urea Nitrogen(BUN); Creatinine(CR); Glucose(GLU); Cholesterol(CHOL); Total Bilirubin(TBIL); Total Protein(TP); Albumin(ALB); Globulin(G); Alanine aminotransferase(ALT); Aspartate aminotransferase(AST); Alkaline phosphatase(ALP); γ-glutamyl transpeptidase(γ-GT)

**Table 16.** Effects of the long-term toxicity test of Wuhu Oral Liquid on the relative weight of major organs in rats (**±SD**)

| Indicator | Time (days) | High-dose group | Low-dose group | Solvent group | Control group |
| --- | --- | --- | --- | --- | --- |
| Heart(g) | D28 | 0.339±0.049 | 0.306±0.022 | 0.314±0.038 | 0.321±0.042 |
|  | D42 | 0.350±0.034 | 0.340±0.046 | 0.341±0.031 | 0.332±0.051 |
| Liver(g) | D28 | 3.506±0.779 | 3.370±0.340 | 3.369±0.597 | 3.217±0.321 |
|  | D42 | 3.270±0.616 | 3.182±0.512 | 3.198±0.496 | 3.112±0.401 |
| Kidney(g) | D28 | 0.757±0.127 | 0.701±0.058 | 0.737±0.066 | 0.716±0.092 |
|  | D42 | 0.752±0.051 | 0.781±0.070 | 0.763±0.081 | 0.770±0.052 |
| Spleen(g) | D28 | 0.395±0.166 | 0.379±0.054 | 0.034±0.046 | 0.359±0.123 |
|  | D42 | 0.322±0.116 | 0.317±0.043 | 0.302±0.035 | 0.327±0.028 |
| Lungs(g) | D28 | 0.711±0.158 | 0.665±0.116 | 0.686±0.141 | 0.709±1.60 |
|  | D42 | 0.687±0.126 | 0.633±0.187 | 0.643±0.118 | 0.702±0.119 |
| Thyroid(mg) | D28 | 7.11±1.90 | 6.77±1.66 | 6.82±1.26 | 6.92±1.33 |
|  | D42 | 6.99±1.72 | 6.82±1.38 | 6.71±1.34 | 6.59±1.80 |
| Adrenal | D28 | 25.01±9.67 | 24.43±4.90 | 22.80±6.66 | 22.60±7.12 |
| gland(mg) | D42 | 25.03±9.00 | 24.89±7.92 | 24.71±6.71 | 23.54±9.03 |
| Prostate(mg) | D28 | 0.346±0.094 | 0.346±0.045 | 0.324±0.073 | 0.298±0.060 |
|  | D42 | 0.300±0.041 | 0.316±0.023 | 0.339±0.051 | 0.312±0.068 |
| Testis(g) | D28 | 1.031±0.090 | 1.146±0.158 | 1.086±0.136 | 1.124±0.181 |
|  | D42 | 1.271±0.132 | 1.192±0.190 | 1.201±0.141 | 1.190±0.201 |
| Ovary(mg) | D28 | 37.18.±5.28 | 35.85±2.11 | 37.28±5.40 | 36.12±5.11 |
|  | D42 | 35.90±3.11 | 33.42±2.13 | 36.72±4.30 | 37.02±3.52 |
| Uterus(g) | D28 | 0.179±0.072 | 0.174±0.053 | 0.150±0.039 | 0.184±0.039 |
|  | D42 | 0.162±0.062 | 0.172±0.060 | 0.618±0.040 | 0.169±0.033 |
| Brain(g) | D28 | 0.481±0.069 | 0.511±0.044 | 0.482±0.087 | 0.472±0.059 |
|  | D42 | 0.492±0.070 | 0.482±0.072 | 0.493±0.076 | 0.497±0.063 |

Note: n=10, Number of sexual organs of animals (n=5)
